# Supplementary material for: NPRL2 gene therapy induces effective antitumor immunity in KRAS/STK11 mutant anti-PD1 resistant metastatic non-small cell lung cancer (NSCLC) in a humanized mouse model
Source: eLife. 2025 Feb 11;13:RP98258. doi: 10.7554/eLife.98258 (PMC11813225; doi:10.7554/eLife.98258)

Figure 1A-source data 1:

PDF of original western blots for Figure 1A, indicating the relevant bands and samples

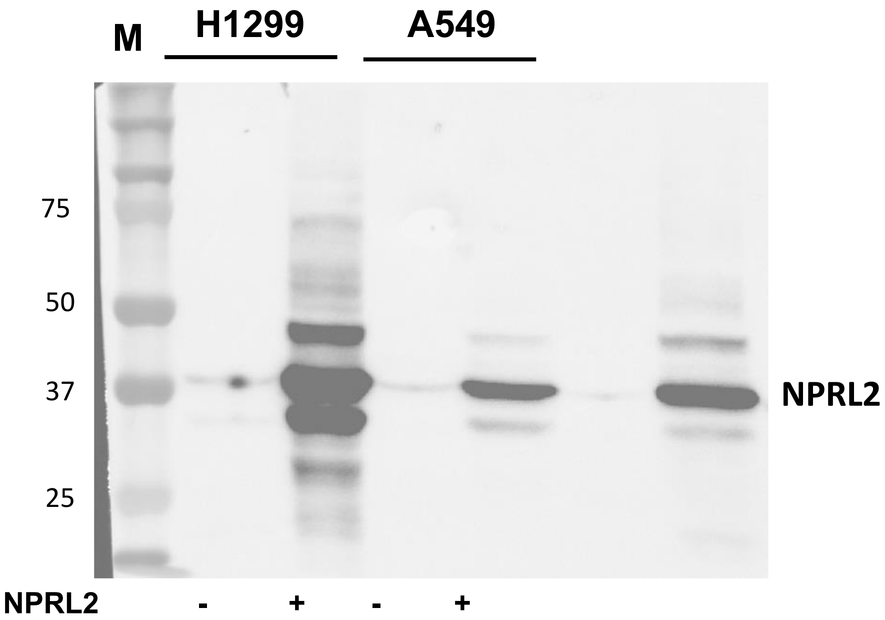

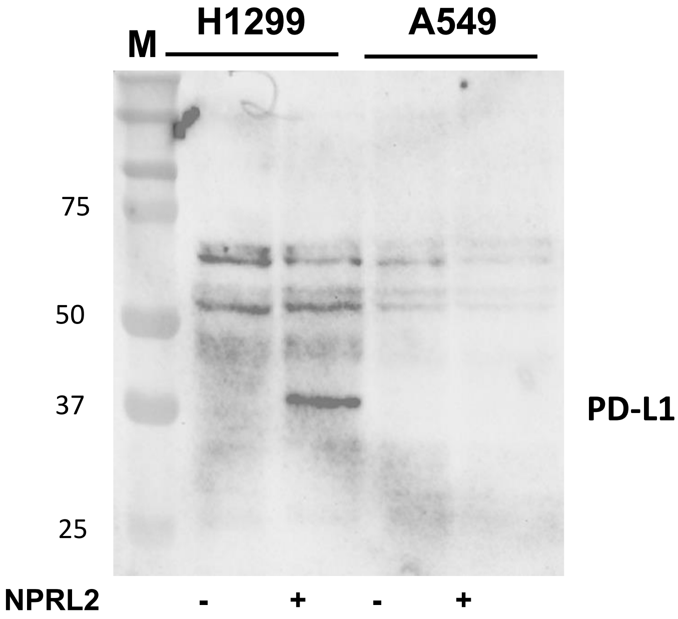

Supplement: Figure 1—source data 2. [file elife-98258-fig1-data2.pdf]
