# Supplementary figures and images for: NPRL2 gene therapy induces effective antitumor immunity in KRAS/STK11 mutant anti-PD1 resistant metastatic non-small cell lung cancer (NSCLC) in a humanized mouse model
Source: eLife. 2025 Feb 11;13:RP98258. doi: 10.7554/eLife.98258 (PMC11813225; doi:10.7554/eLife.98258)

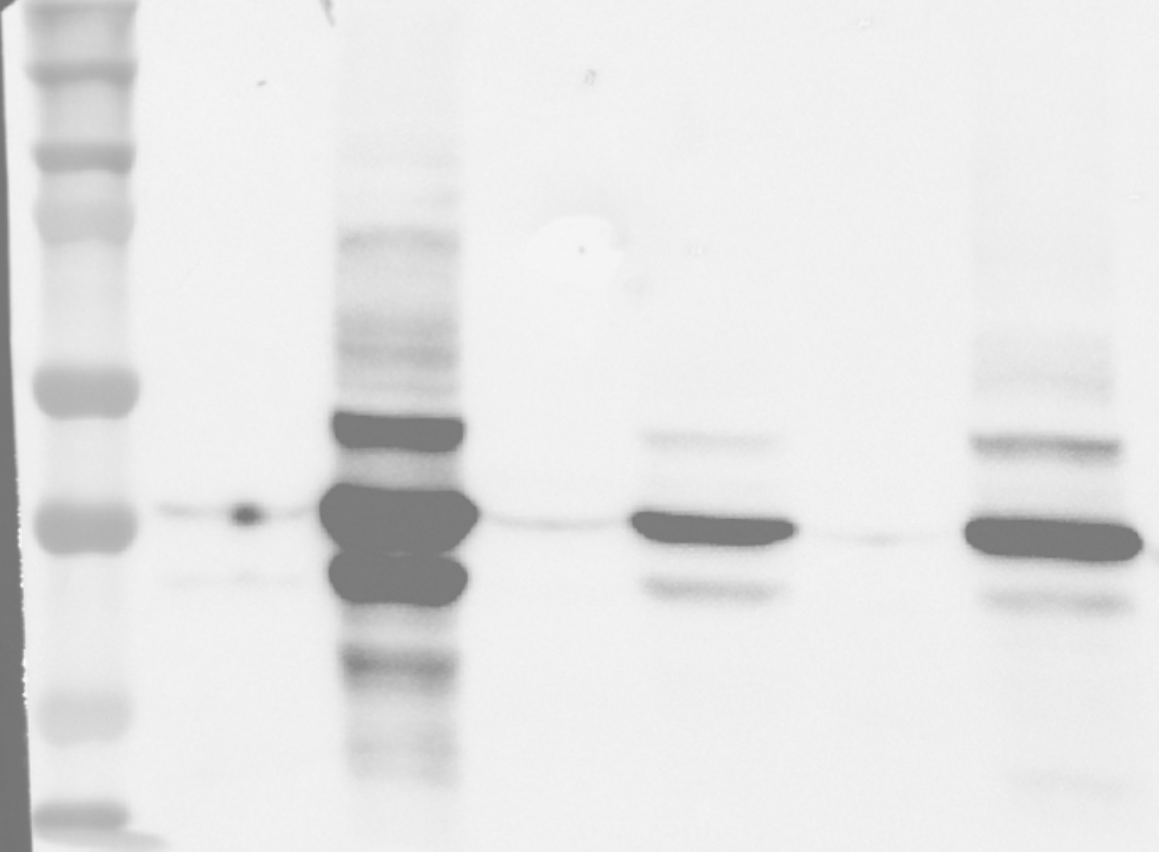

Supplement: Figure 1—source data 3. [file elife-98258-fig1-data3.zip › Fig 1A_Original WB_ TIFFs/Figure 1-source data 2. Original files for western blot analysis displayed in Figure 1A-NPRL2.tif]

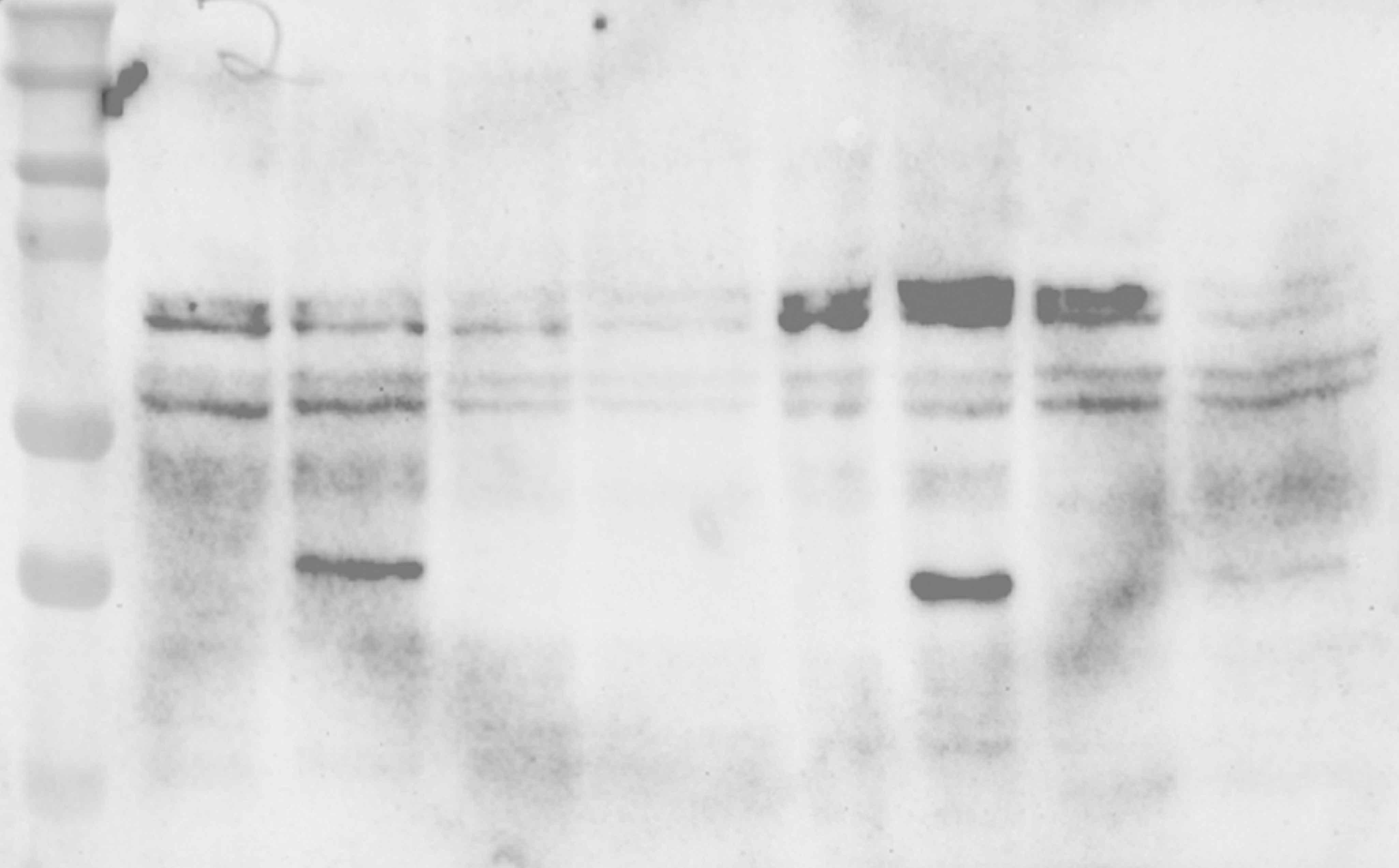

Supplement: Figure 1—source data 3. [file elife-98258-fig1-data3.zip › Fig 1A_Original WB_ TIFFs/Figure 1-source data 2. Original files for western blot analysis displayed in Figure 1A-PDL-1.tif]

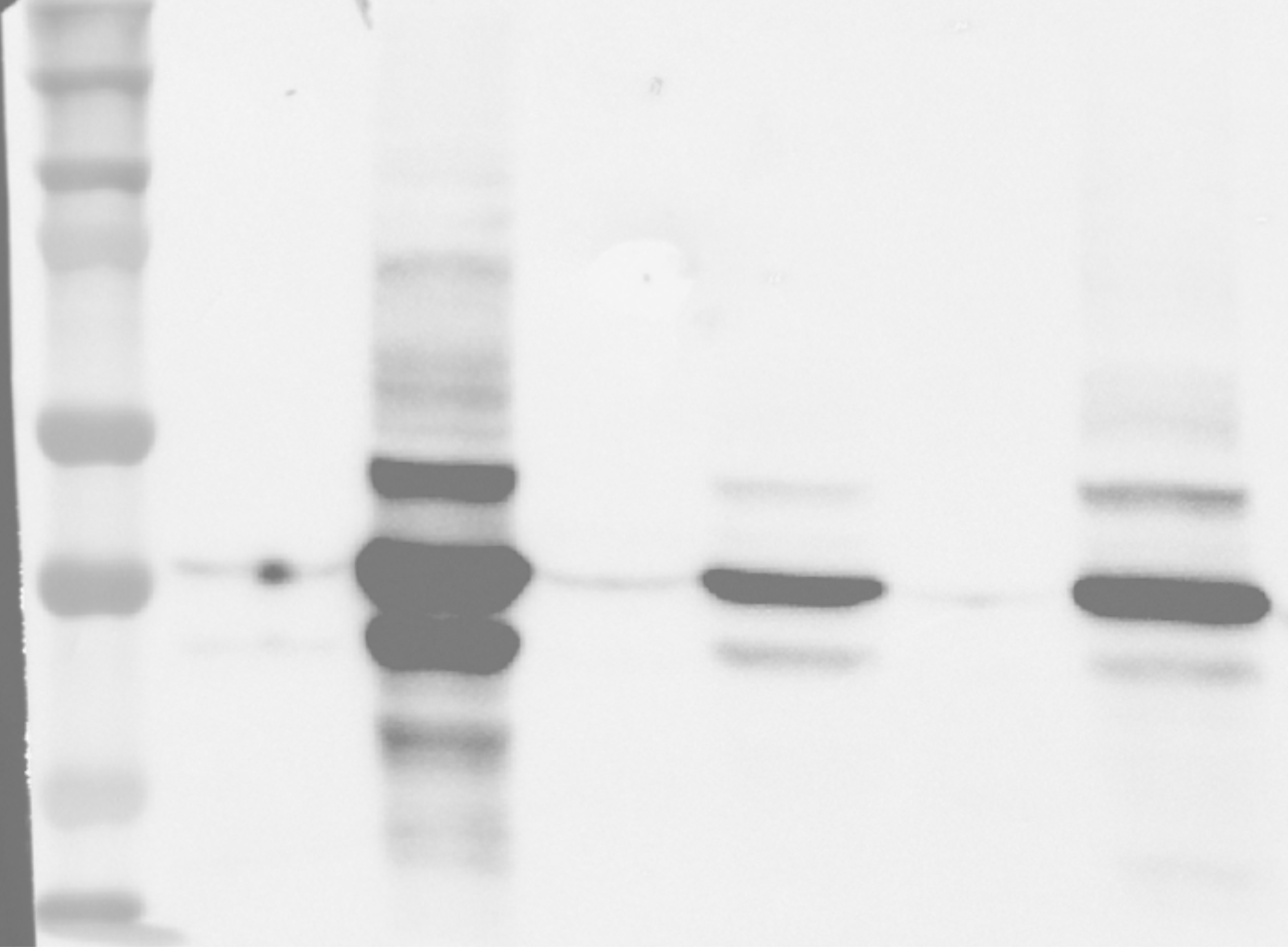

Supplement: Figure 1—figure supplement 1—source data 2. [file elife-98258-fig1-figsupp1-data2.zip › Fig 1-fig suppl 1_Original WB_TIFFs/Fig 1-fig suppl 1-Original WB images-H1299 A549 H358.tif]

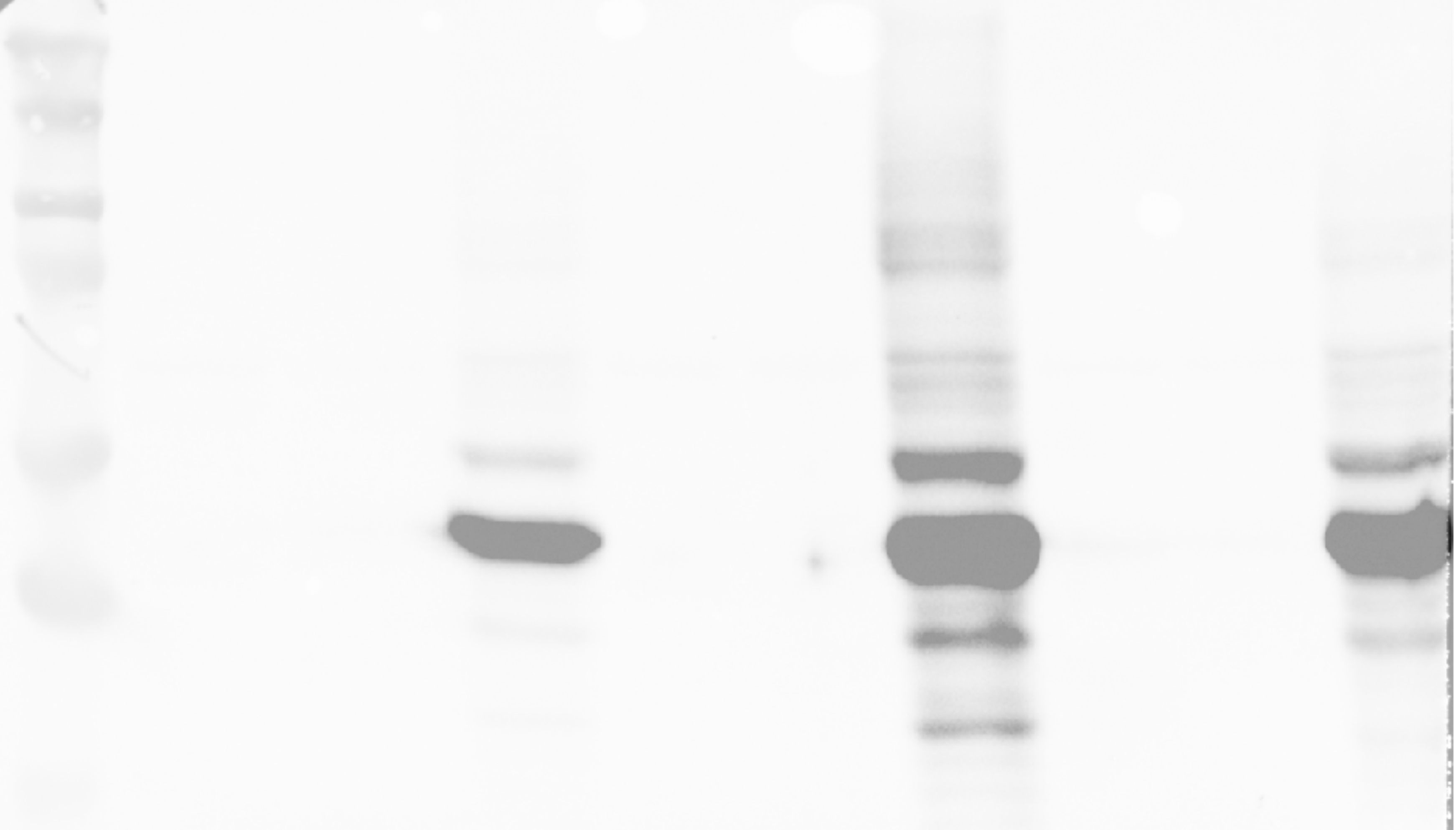

Supplement: Figure 1—figure supplement 1—source data 2. [file elife-98258-fig1-figsupp1-data2.zip › Fig 1-fig suppl 1_Original WB_TIFFs/Fig 1-fig suppl 1-Original WB images-H1975 H1975R A549.tif]

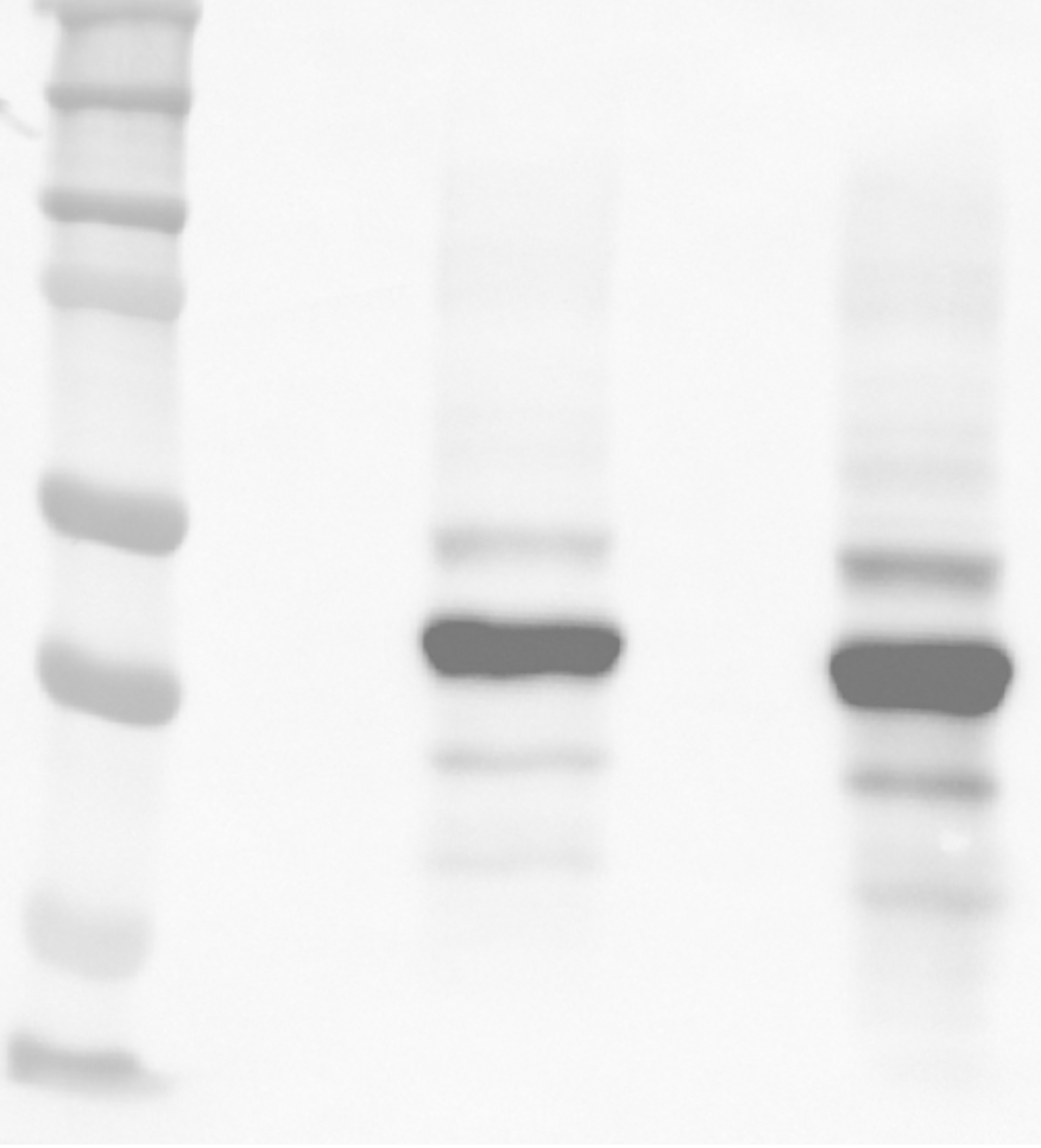

Supplement: Figure 1—figure supplement 1—source data 2. [file elife-98258-fig1-figsupp1-data2.zip › Fig 1-fig suppl 1_Original WB_TIFFs/Fig 1-fig suppl 1-Original WB images-H23 H23AR.tif]

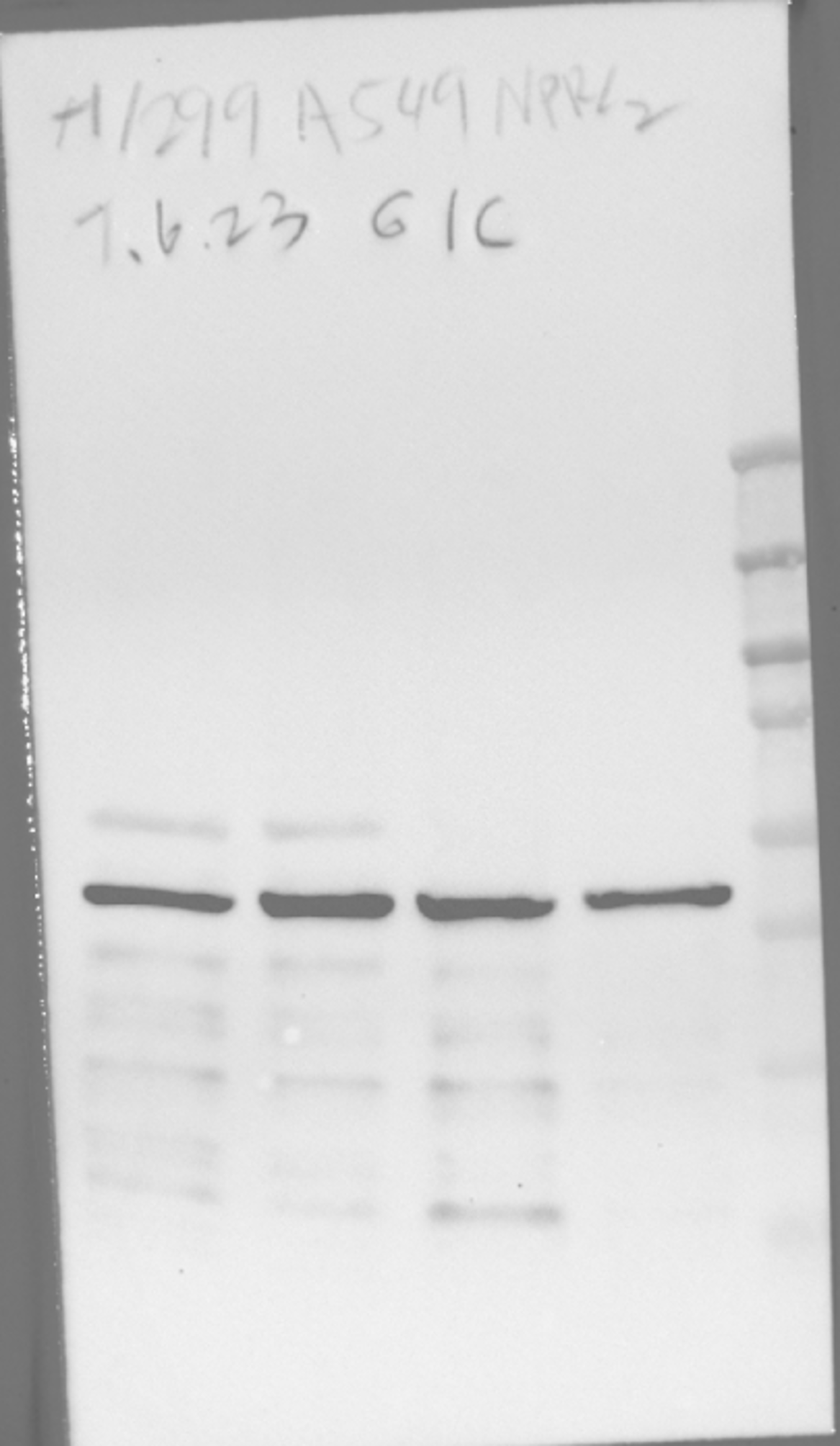

Supplement: Figure 8—source data 3. [file elife-98258-fig8-data3.zip › Figure 8-source data 2/Figure 8-source data 2-b-actin.tif]

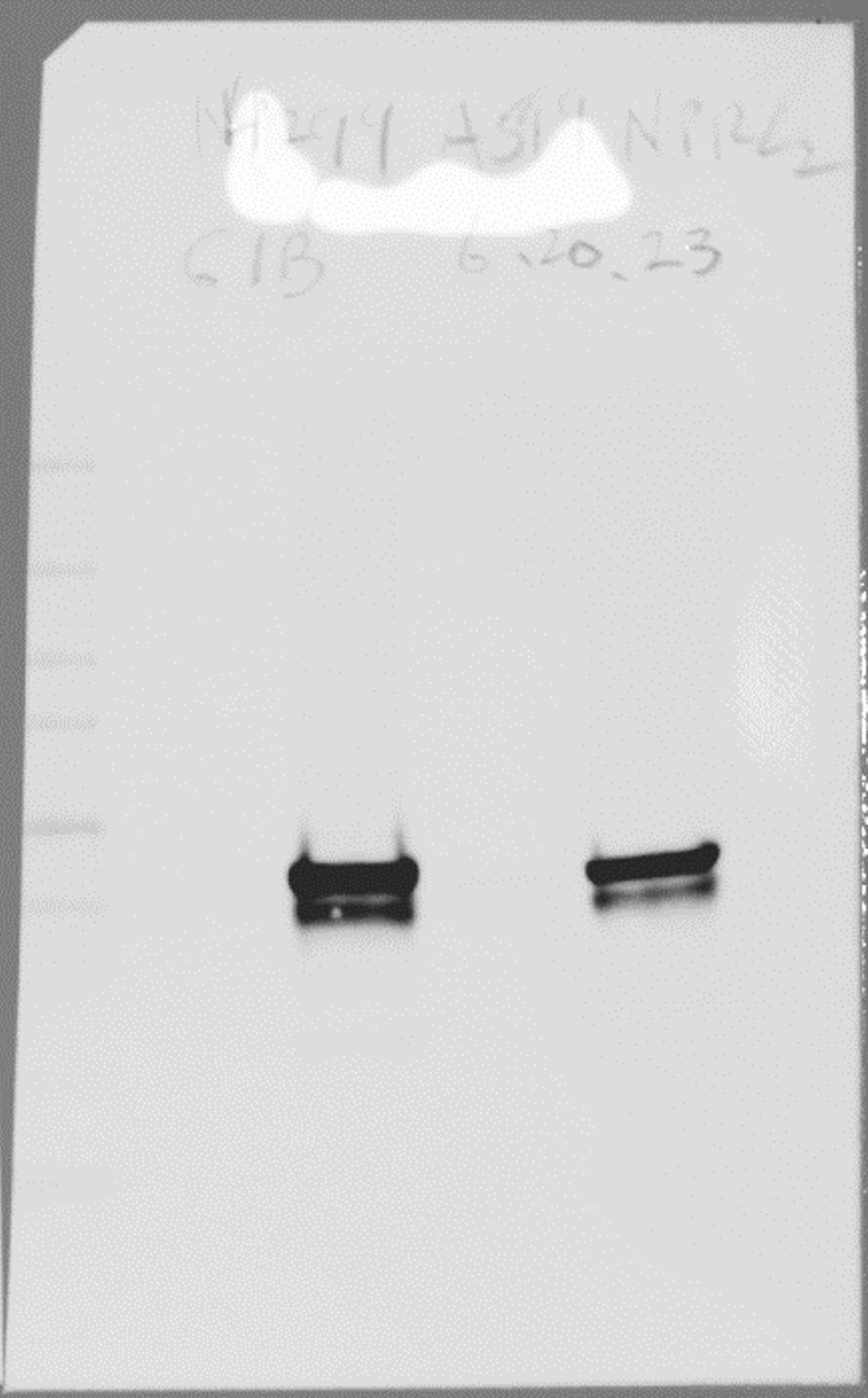

Supplement: Figure 8—source data 3. [file elife-98258-fig8-data3.zip › Figure 8-source data 2/Figure 8-source data 2-NPRL2.tif]

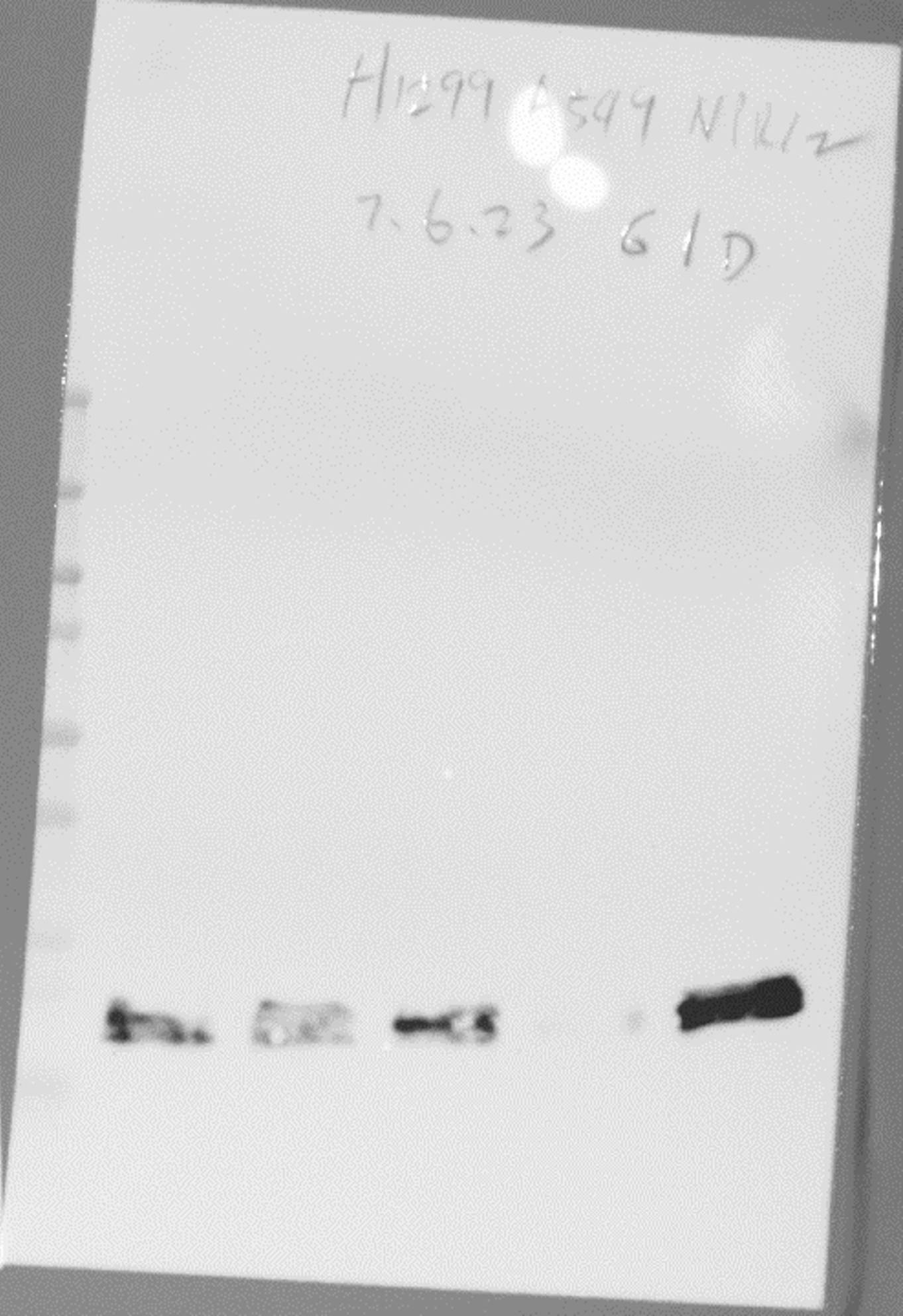

Supplement: Figure 8—source data 3. [file elife-98258-fig8-data3.zip › Figure 8-source data 2/Figure 8-source data 2-p4E-BP1.tif]

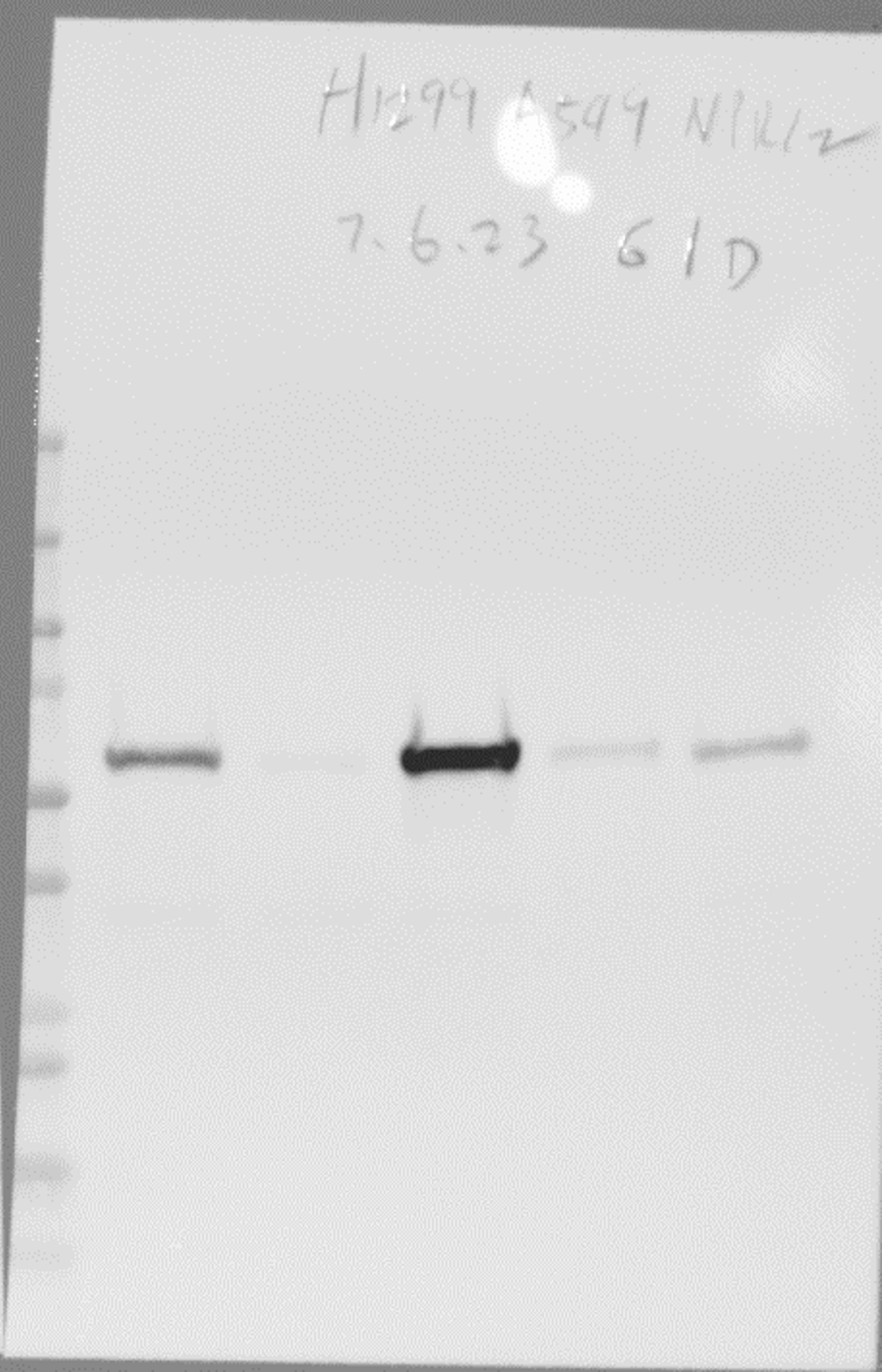

Supplement: Figure 8—source data 3. [file elife-98258-fig8-data3.zip › Figure 8-source data 2/Figure 8-source data 2-pAKT.tif]

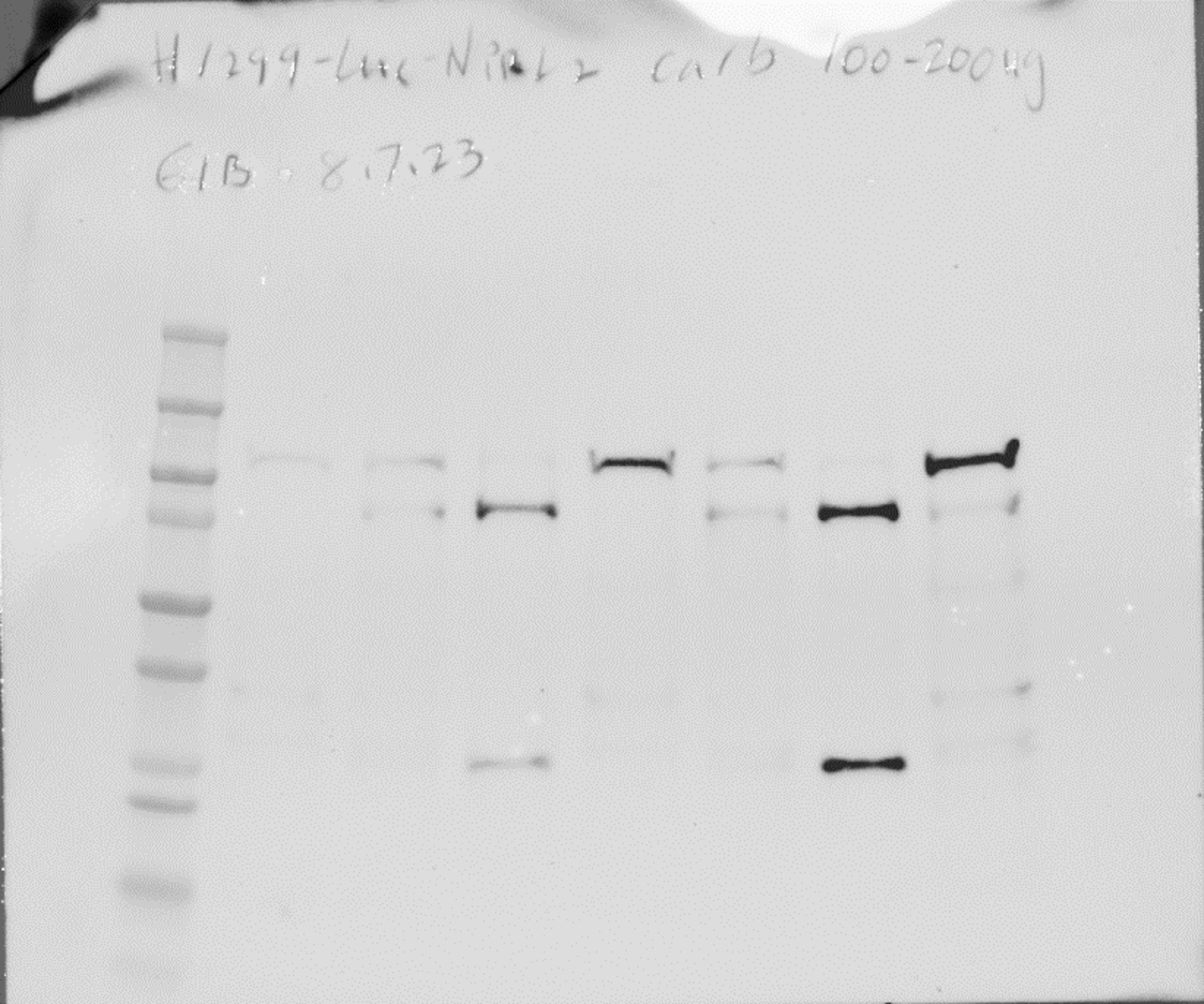

Supplement: Figure 8—source data 3. [file elife-98258-fig8-data3.zip › Figure 8-source data 2/Figure 8-source data 2-PARP Cleavage 1.tif]

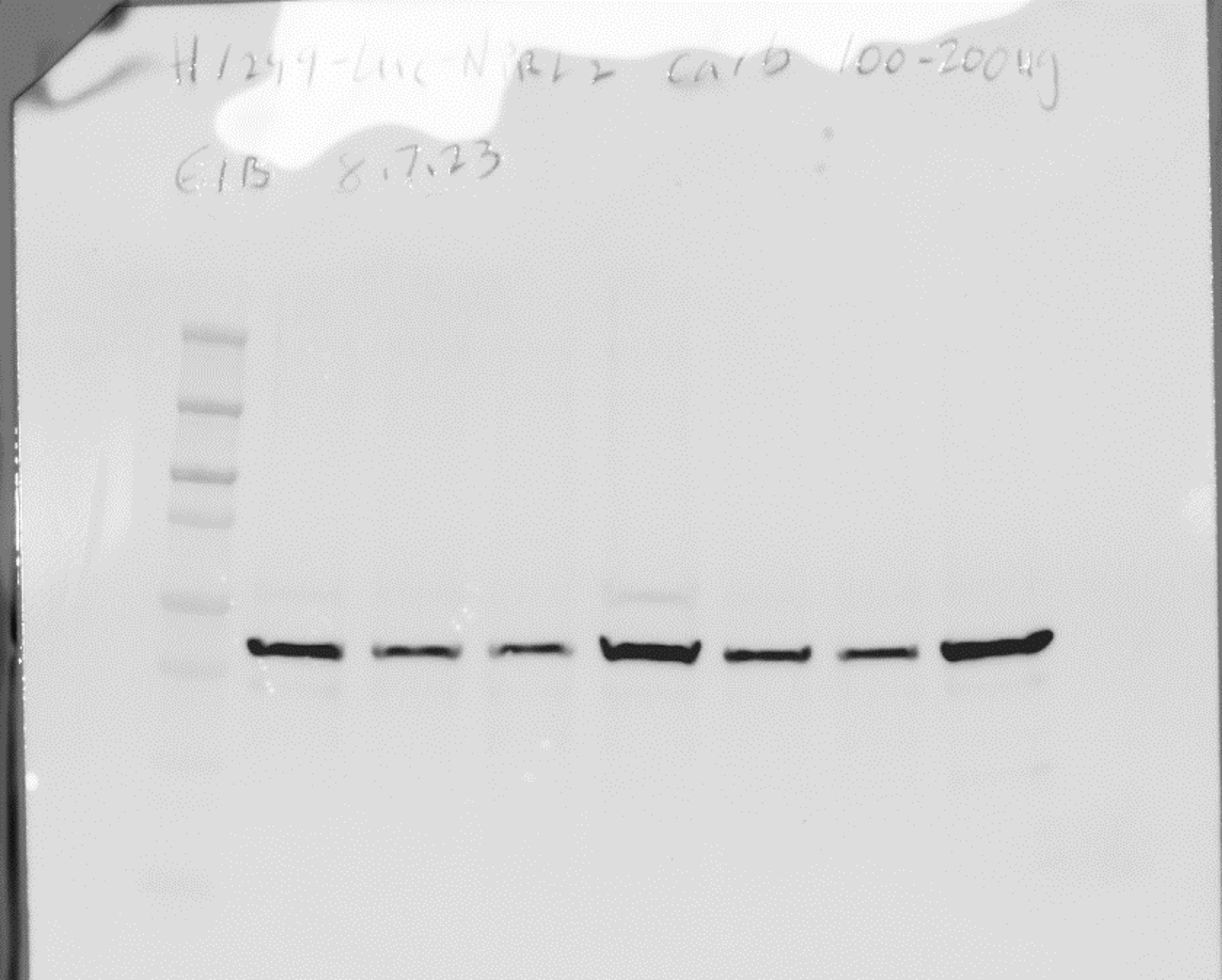

Supplement: Figure 8—source data 3. [file elife-98258-fig8-data3.zip › Figure 8-source data 2/Figure 8-source data 2-PARP Cleavage 1-b-actin.tif]

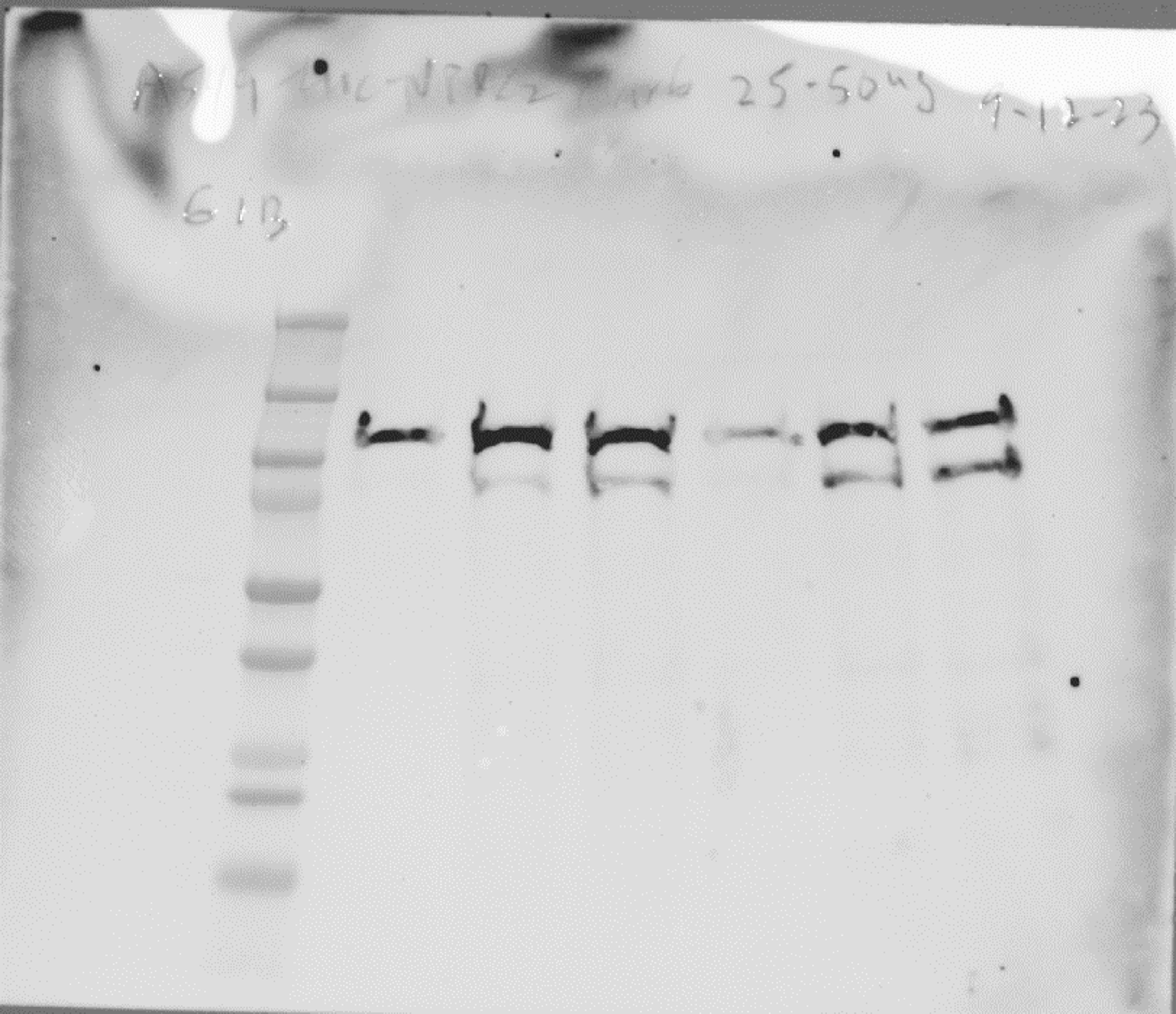

Supplement: Figure 8—source data 3. [file elife-98258-fig8-data3.zip › Figure 8-source data 2/Figure 8-source data 2-PARP Cleavage 2.tif]

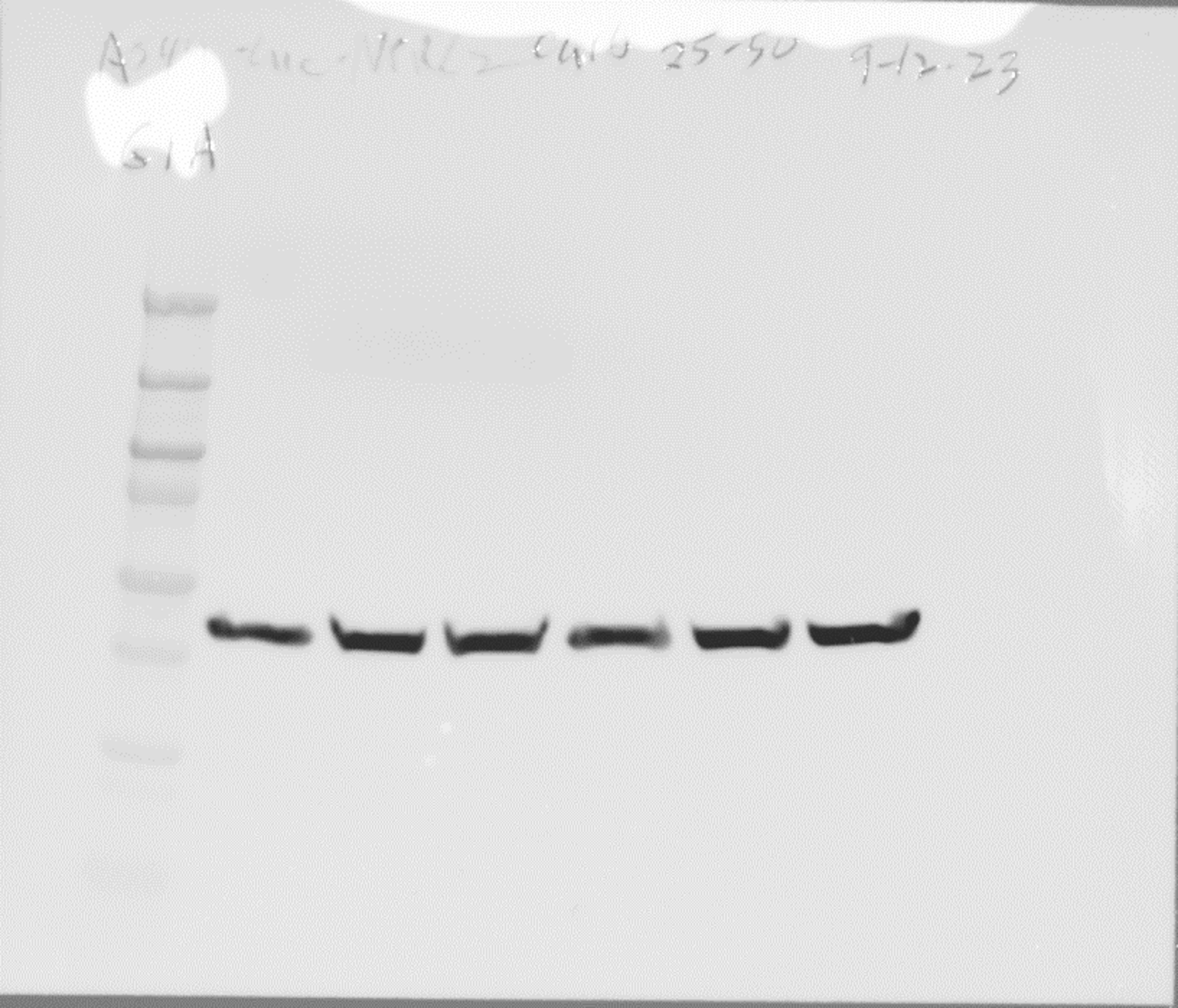

Supplement: Figure 8—source data 3. [file elife-98258-fig8-data3.zip › Figure 8-source data 2/Figure 8-source data 2-PARP Cleavage 2-b-actin.tif]

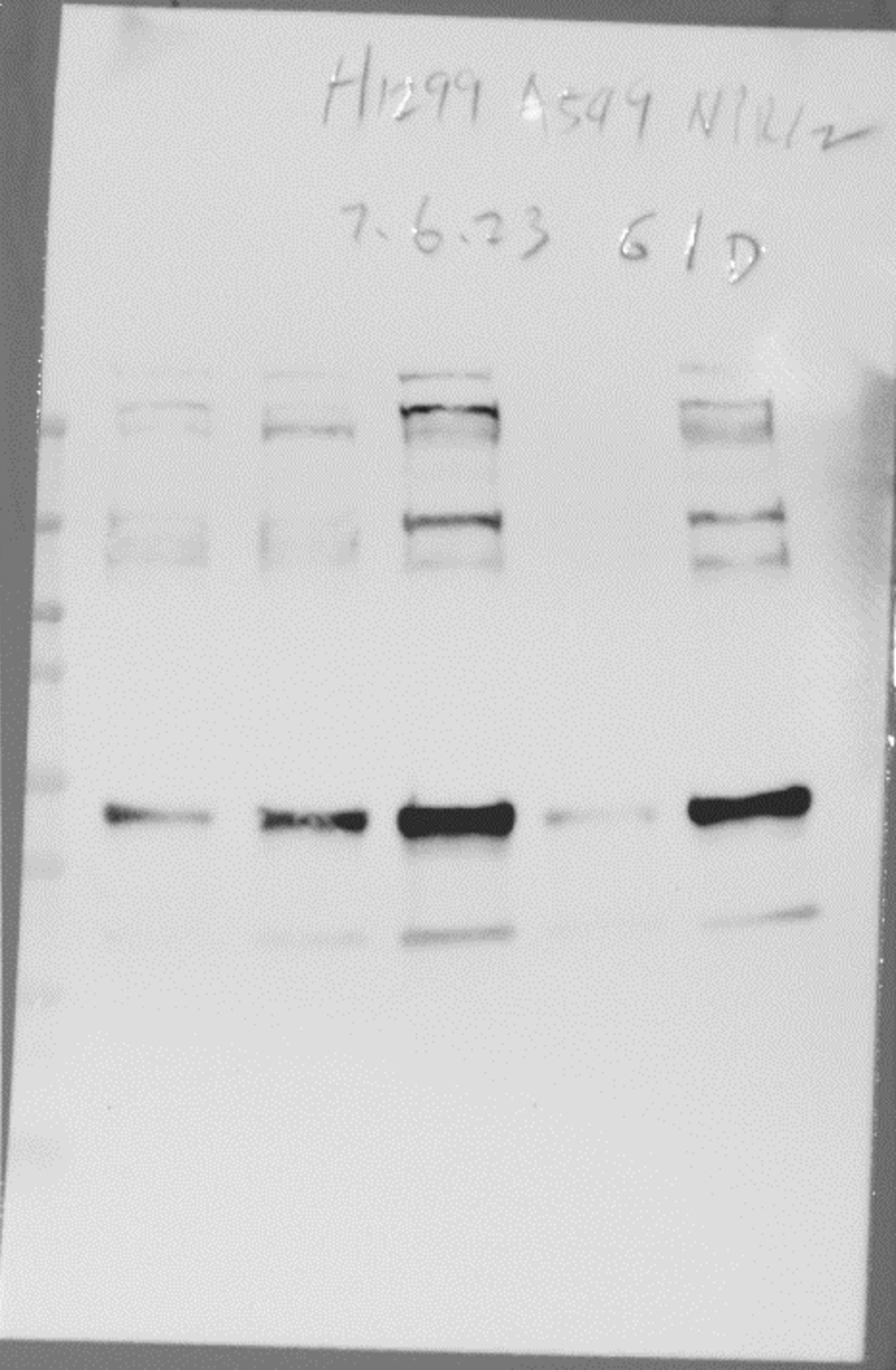

Supplement: Figure 8—source data 3. [file elife-98258-fig8-data3.zip › Figure 8-source data 2/Figure 8-source data 2-pGSK-3b.tif]

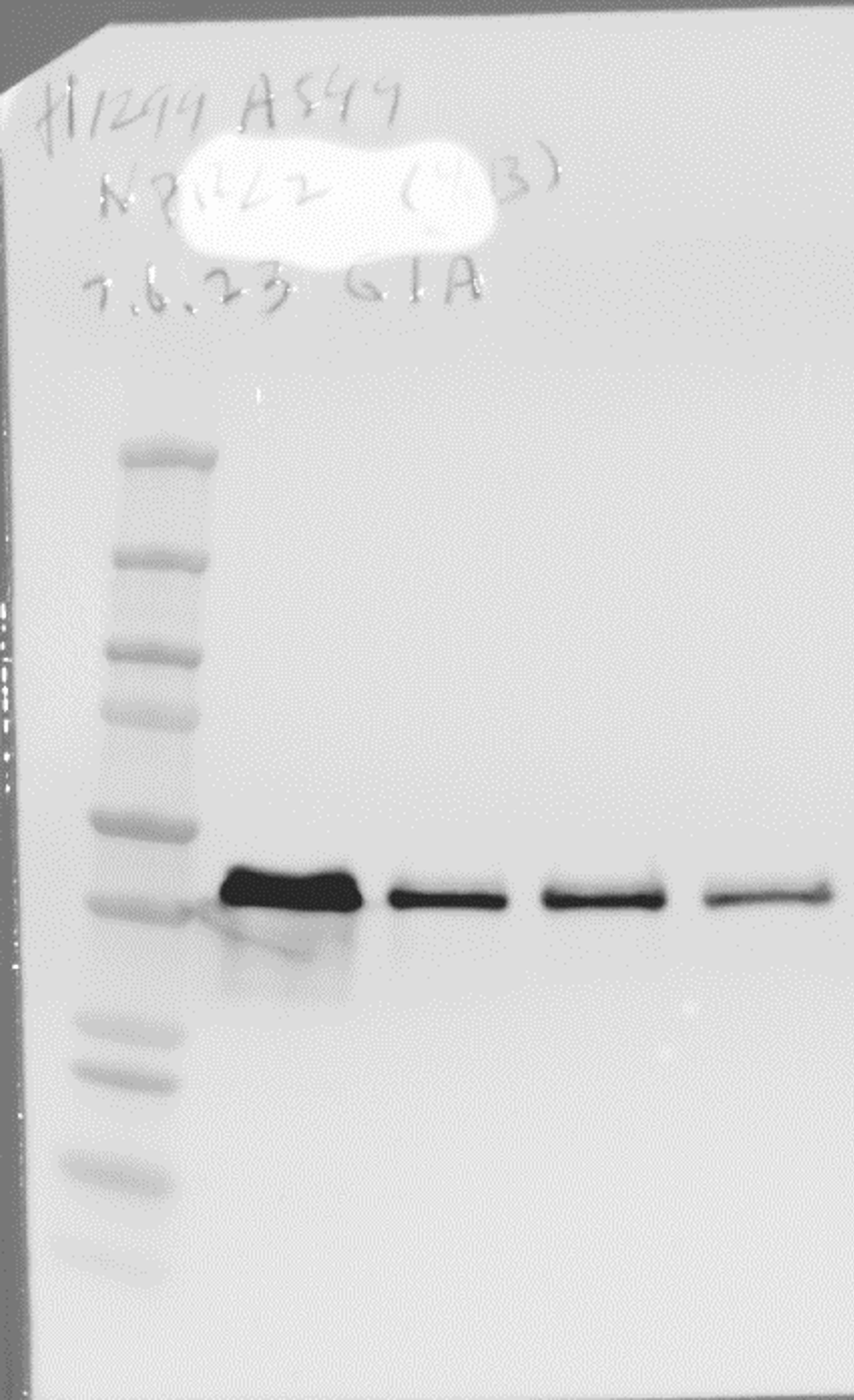

Supplement: Figure 8—source data 3. [file elife-98258-fig8-data3.zip › Figure 8-source data 2/Figure 8-source data 2-pMAPK.tif]

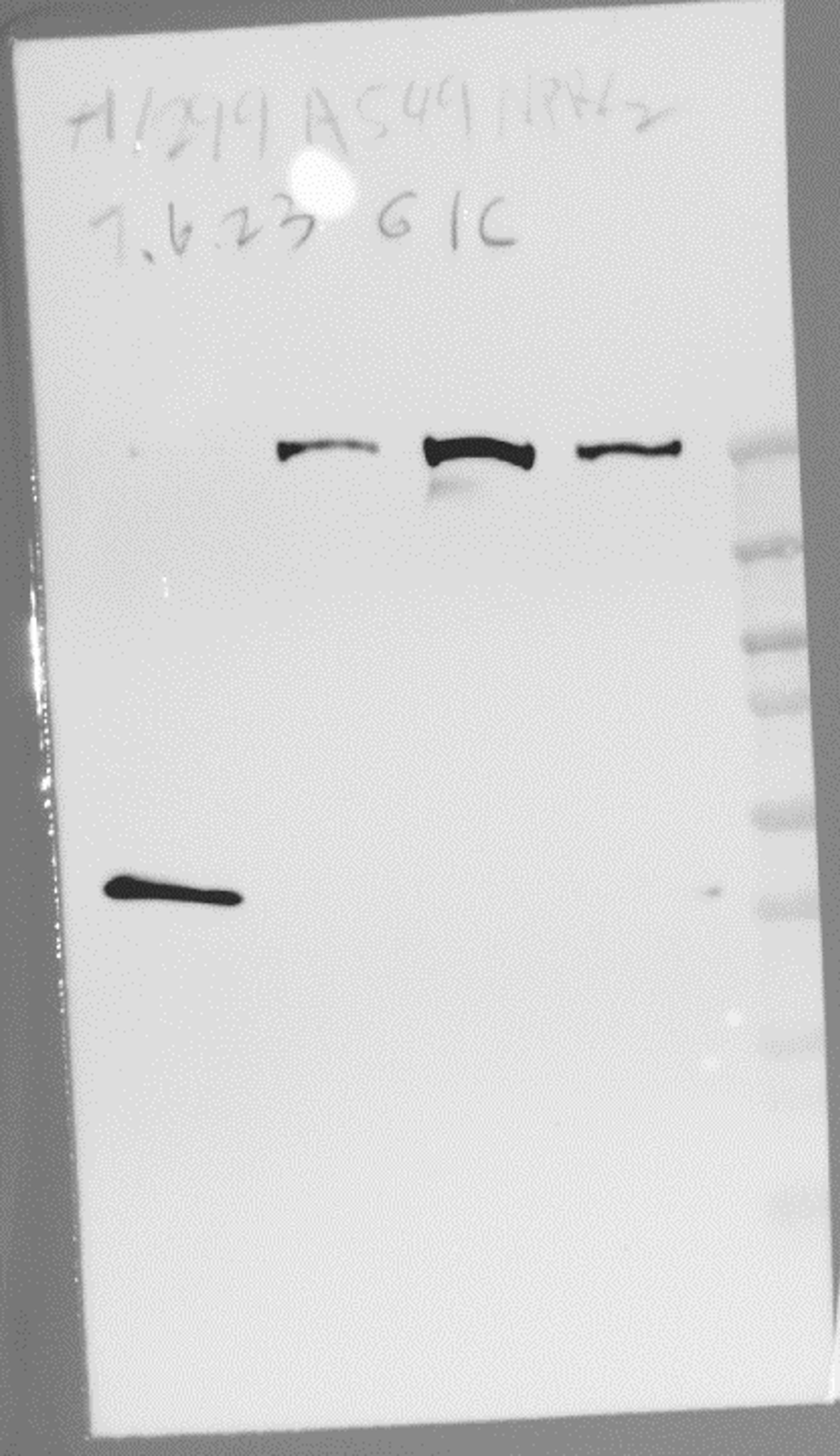

Supplement: Figure 8—source data 3. [file elife-98258-fig8-data3.zip › Figure 8-source data 2/Figure 8-source data 2-pmTOR.tif]

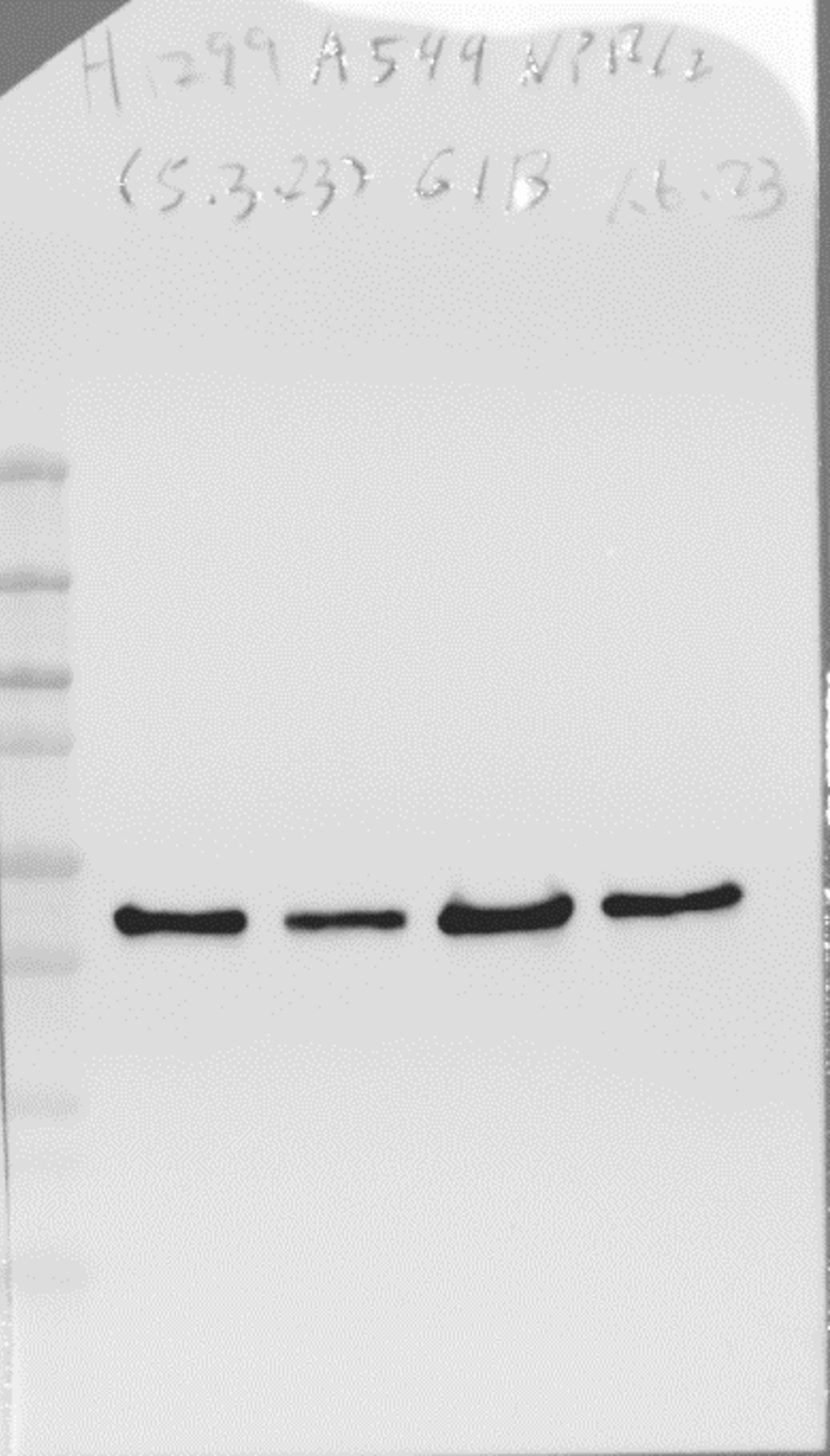

Supplement: Figure 8—source data 3. [file elife-98258-fig8-data3.zip › Figure 8-source data 2/Figure 8-source data 2-pPRAS40.tif]

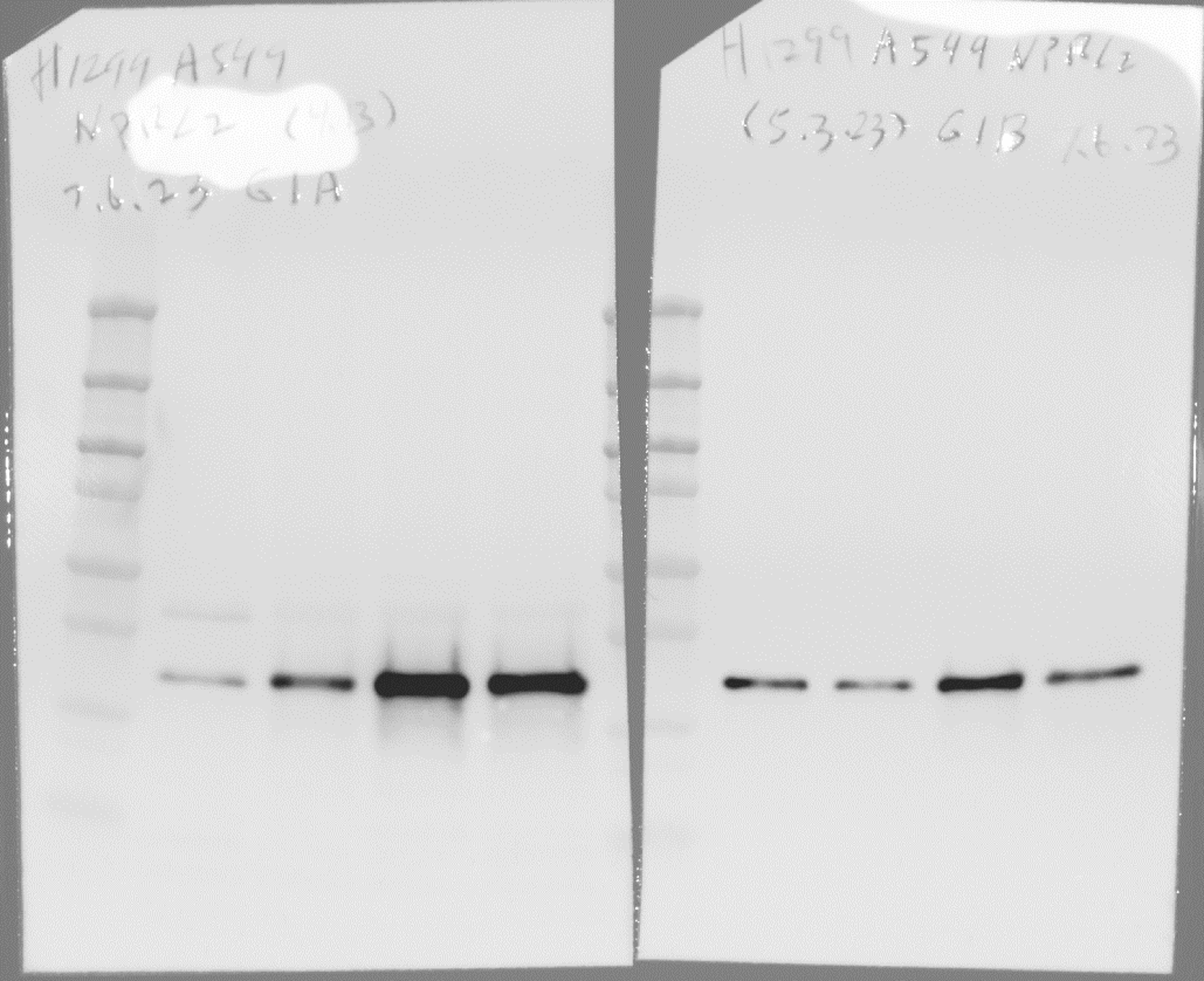

Supplement: Figure 8—source data 3. [file elife-98258-fig8-data3.zip › Figure 8-source data 2/Figure 8-source data 2-pS6.tif]
