# Supplementary material for: NPRL2 gene therapy induces effective antitumor immunity in KRAS/STK11 mutant anti-PD1 resistant metastatic non-small cell lung cancer (NSCLC) in a humanized mouse model
Source: eLife. 2025 Feb 11;13:RP98258. doi: 10.7554/eLife.98258 (PMC11813225; doi:10.7554/eLife.98258)
Supplement: Figure 1—figure supplement 1—source data 1. [file elife-98258-fig1-figsupp1-data1.pdf]

Figure 1-figure supplement 1-source data 1

PDF file containing original western blots for Figure 1-figure supplement 1, indicating the NPRL2 expressions in different NSCLC cell lines

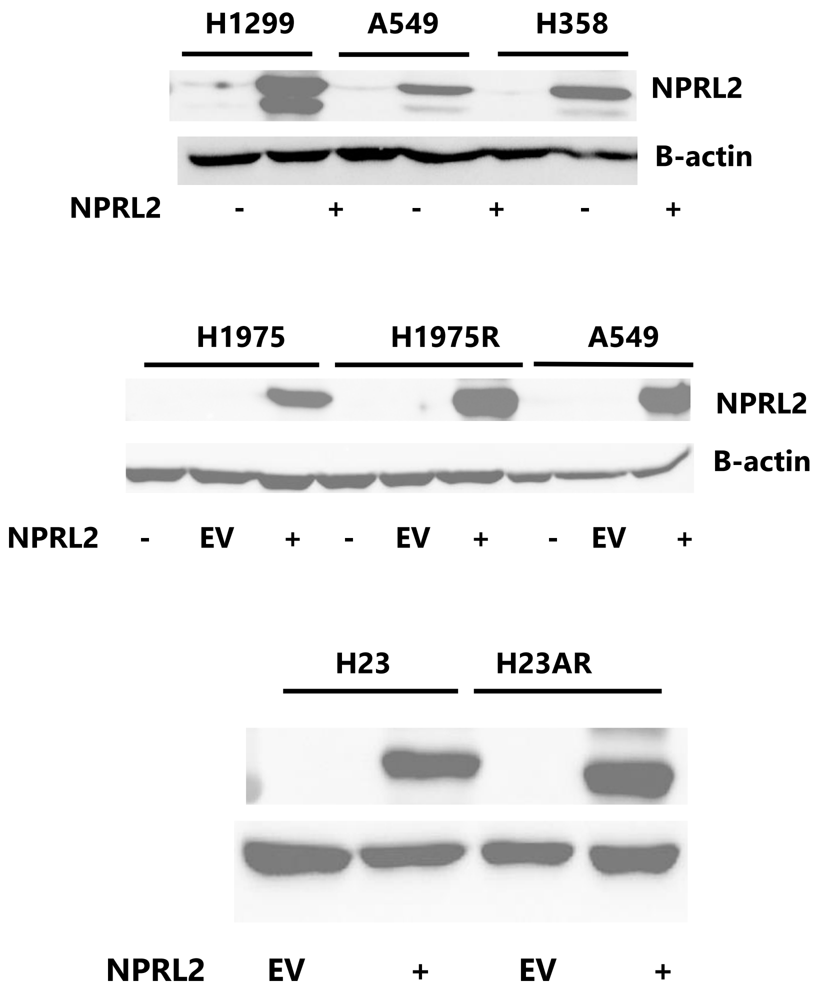

Figure 1-figure supplement 1. Basal level of NPRL2 expression and its transfection on various NSCLC cell lines

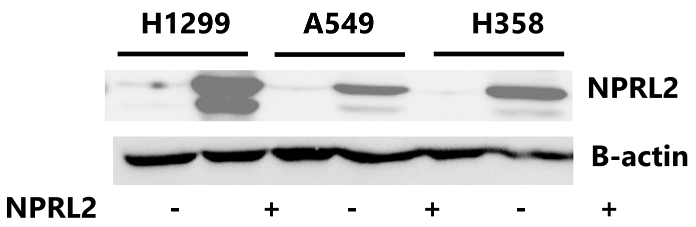

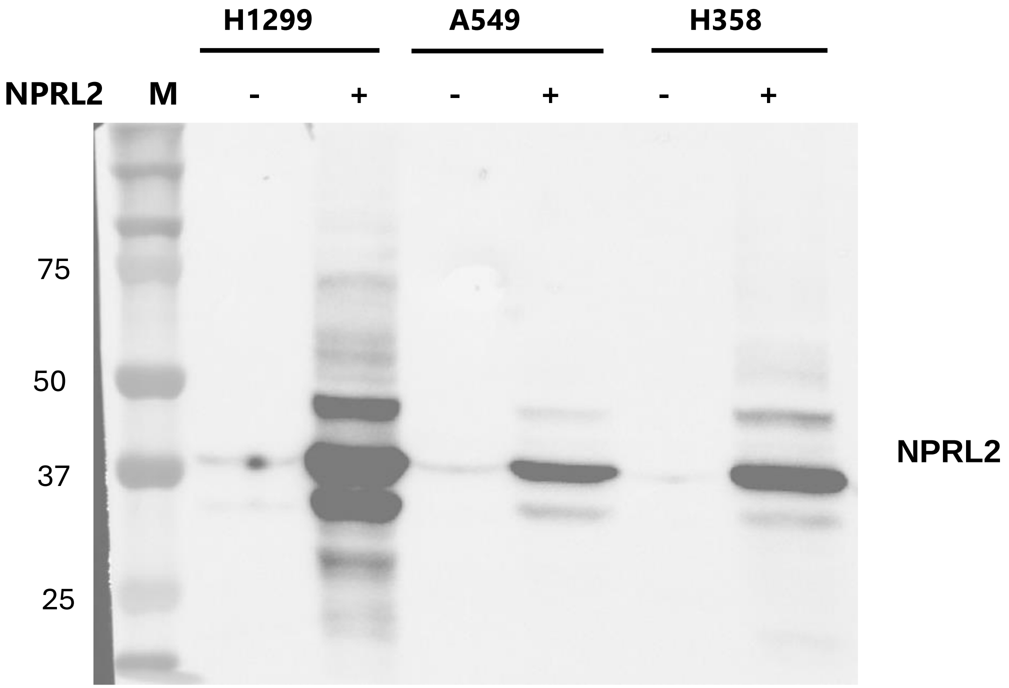

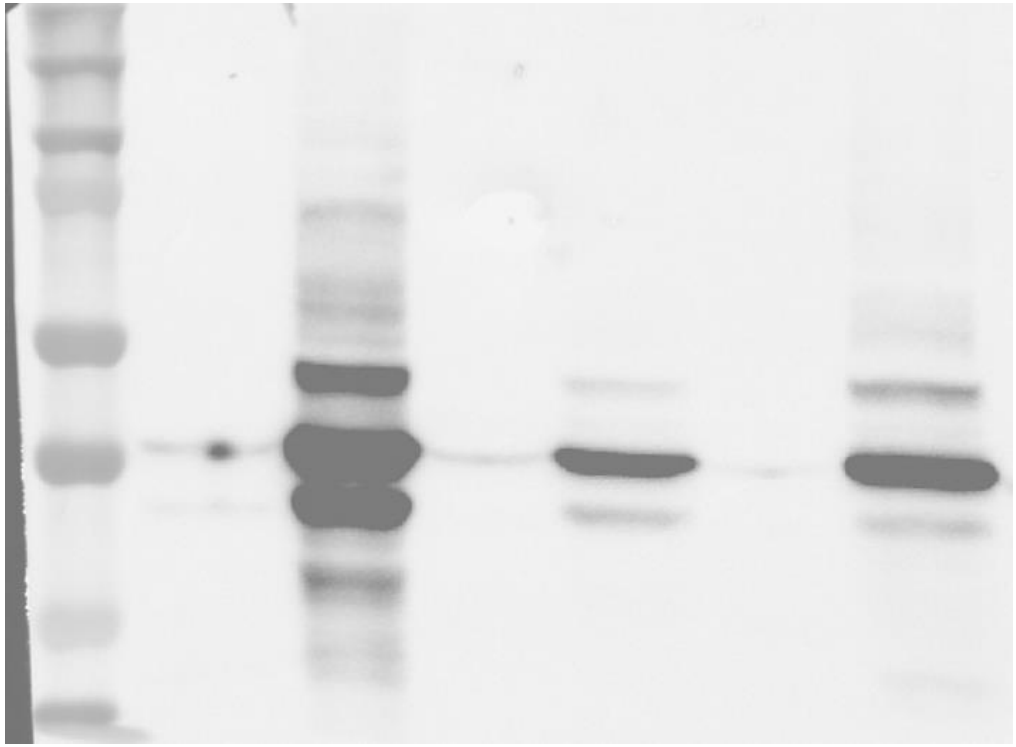

Figure 1-figure Supplement 1-source data 1. Basal level of NPRL2 expression and its transfection on various NSCLC cell lines

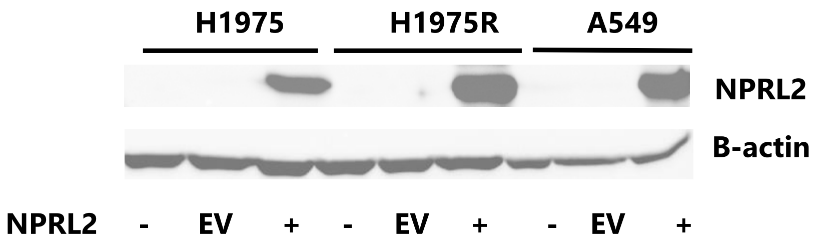

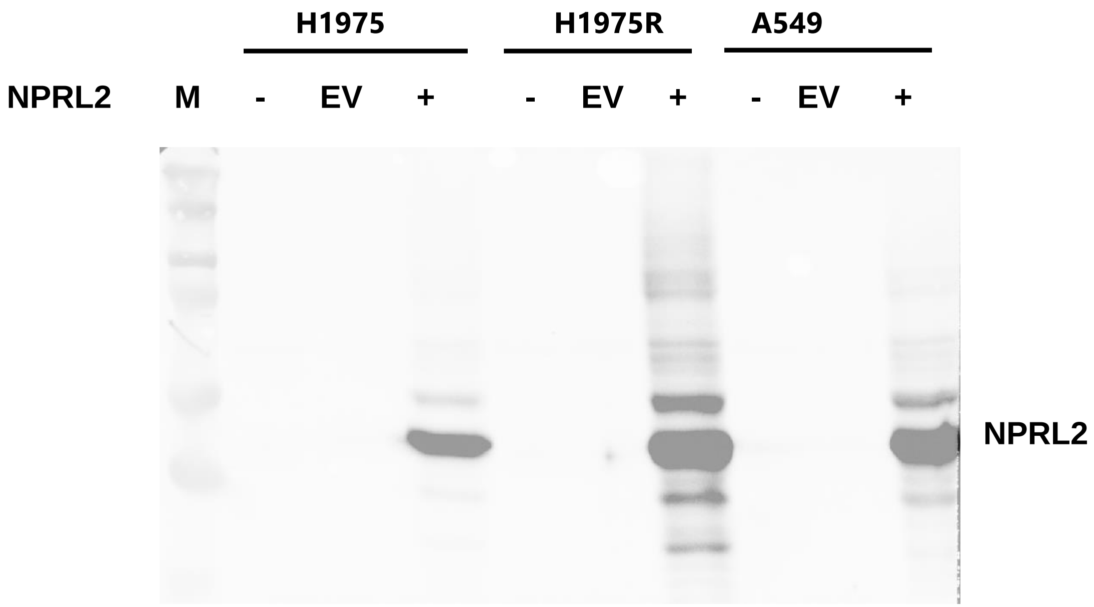

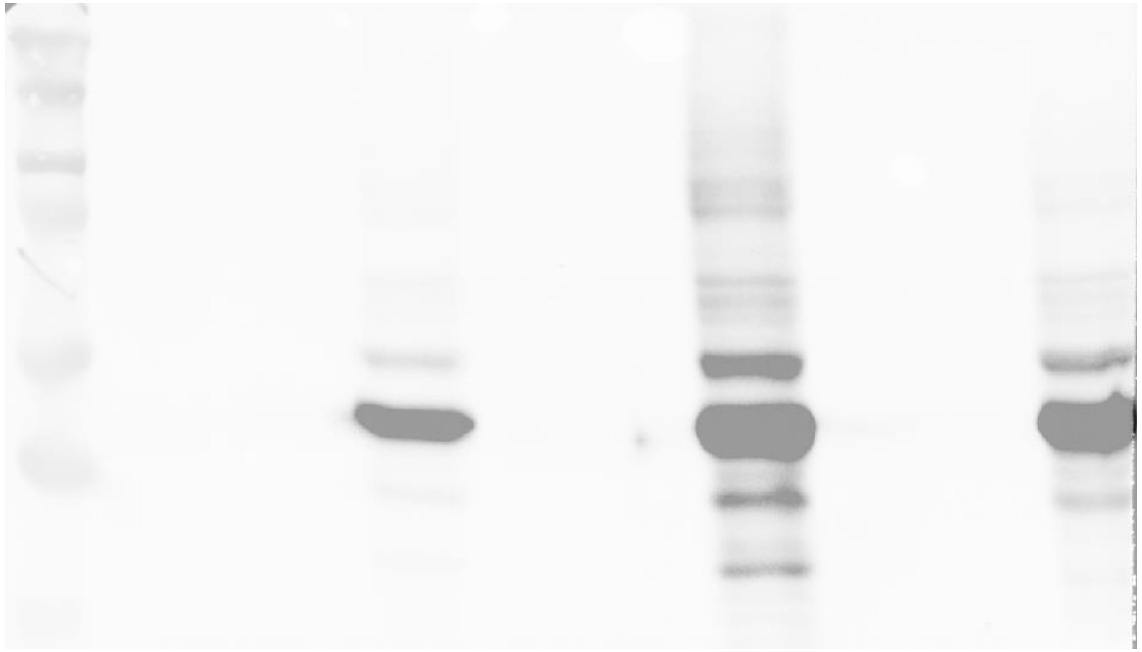

Figure 1-figure Supplement 1-source data 1. Basal level of NPRL2 expression and its transfection on various NSCLC cell lines

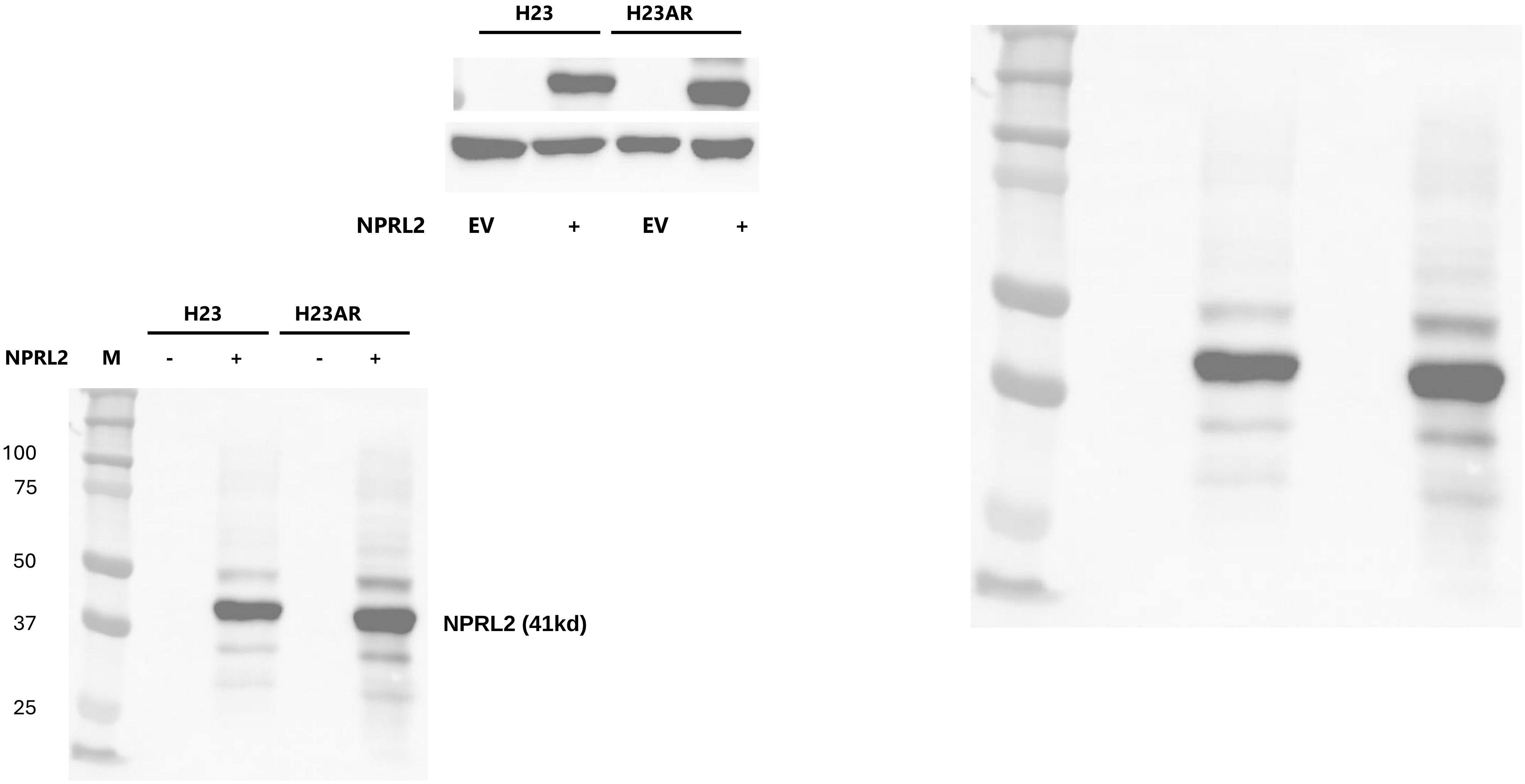

Figure 1-figure Supplement 1-source data 1. Basal level of NPRL2 expression and its transfection on various NSCLC cell lines
