# Supplementary material for: NPRL2 gene therapy induces effective antitumor immunity in KRAS/STK11 mutant anti-PD1 resistant metastatic non-small cell lung cancer (NSCLC) in a humanized mouse model
Source: eLife. 2025 Feb 11;13:RP98258. doi: 10.7554/eLife.98258 (PMC11813225; doi:10.7554/eLife.98258)
Supplement: Figure 8—source data 2. [file elife-98258-fig8-data2.pdf]

Figure 8-source data 1

PDF file containing original western blots for figure 8R and figure 8S, indicating relevant bands, treatments and samples

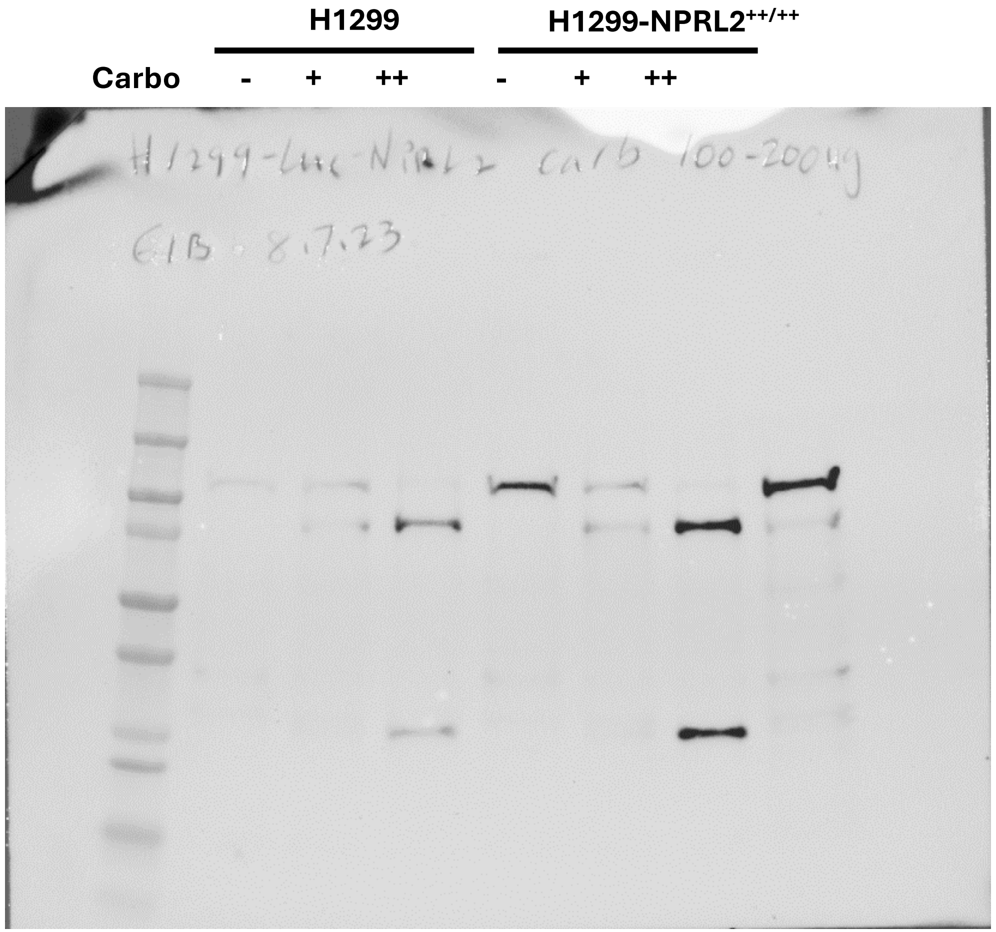

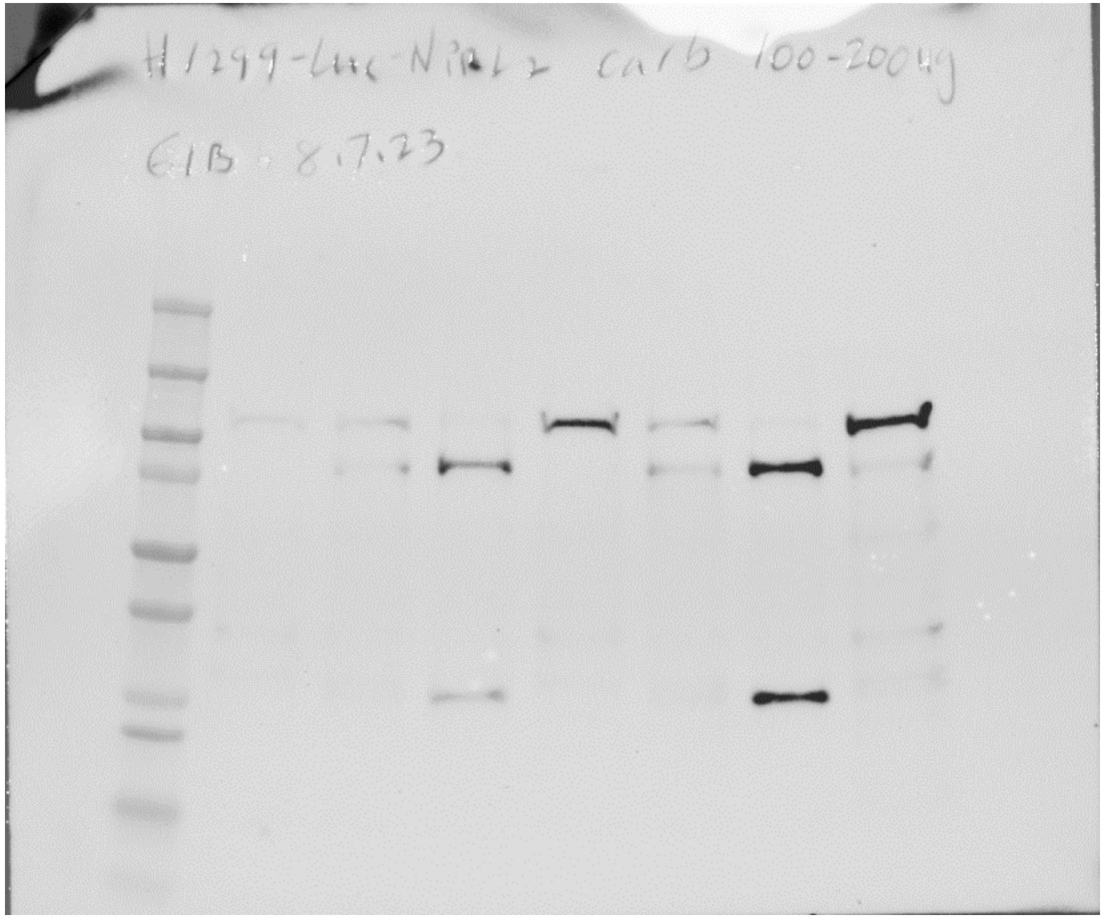

Figure 8-source data 1: PDF file containing original western blots for Figure 8R, indicating the relevant bands and samples

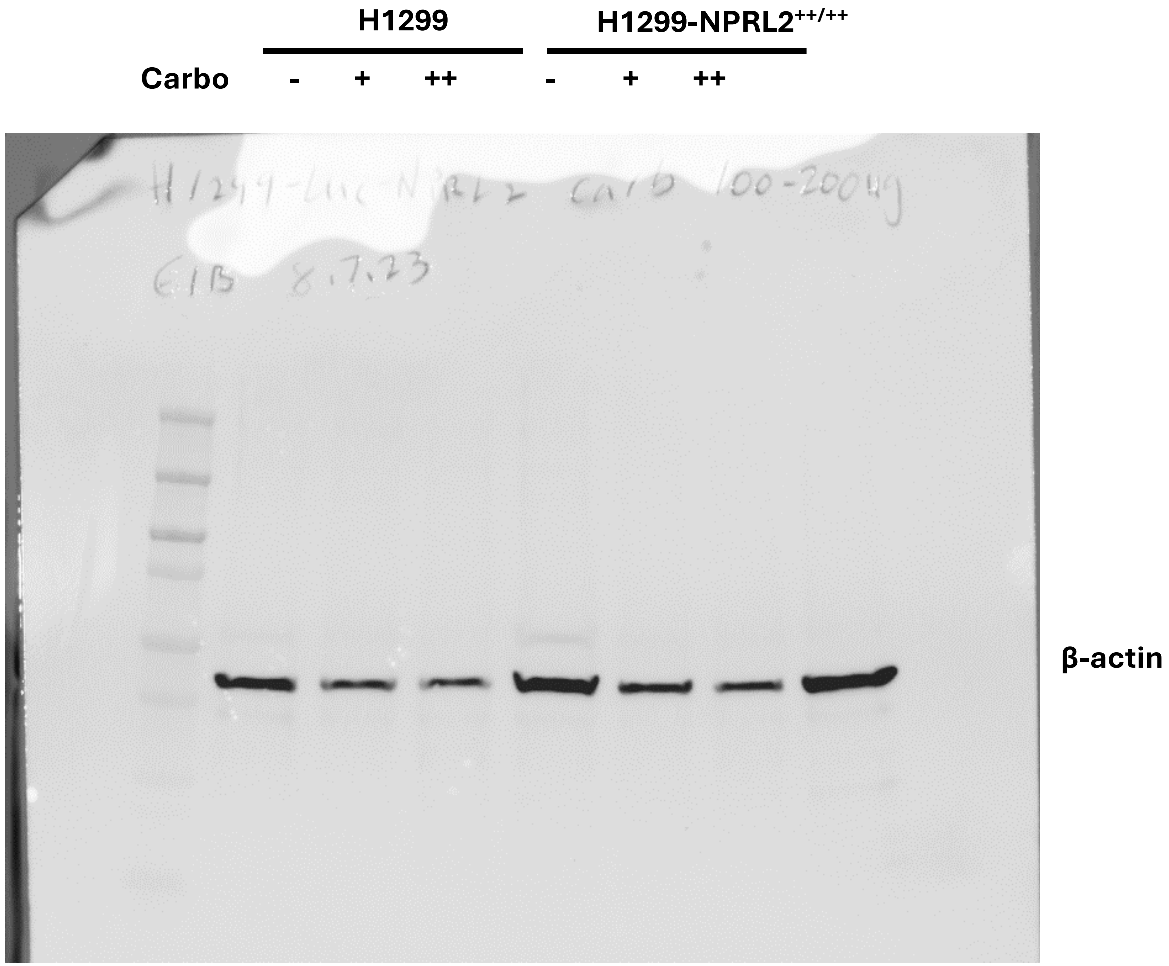

Figure 8-source data 1: PDF file containing original western blots for Figure 8R, indicating the relevant bands and samples

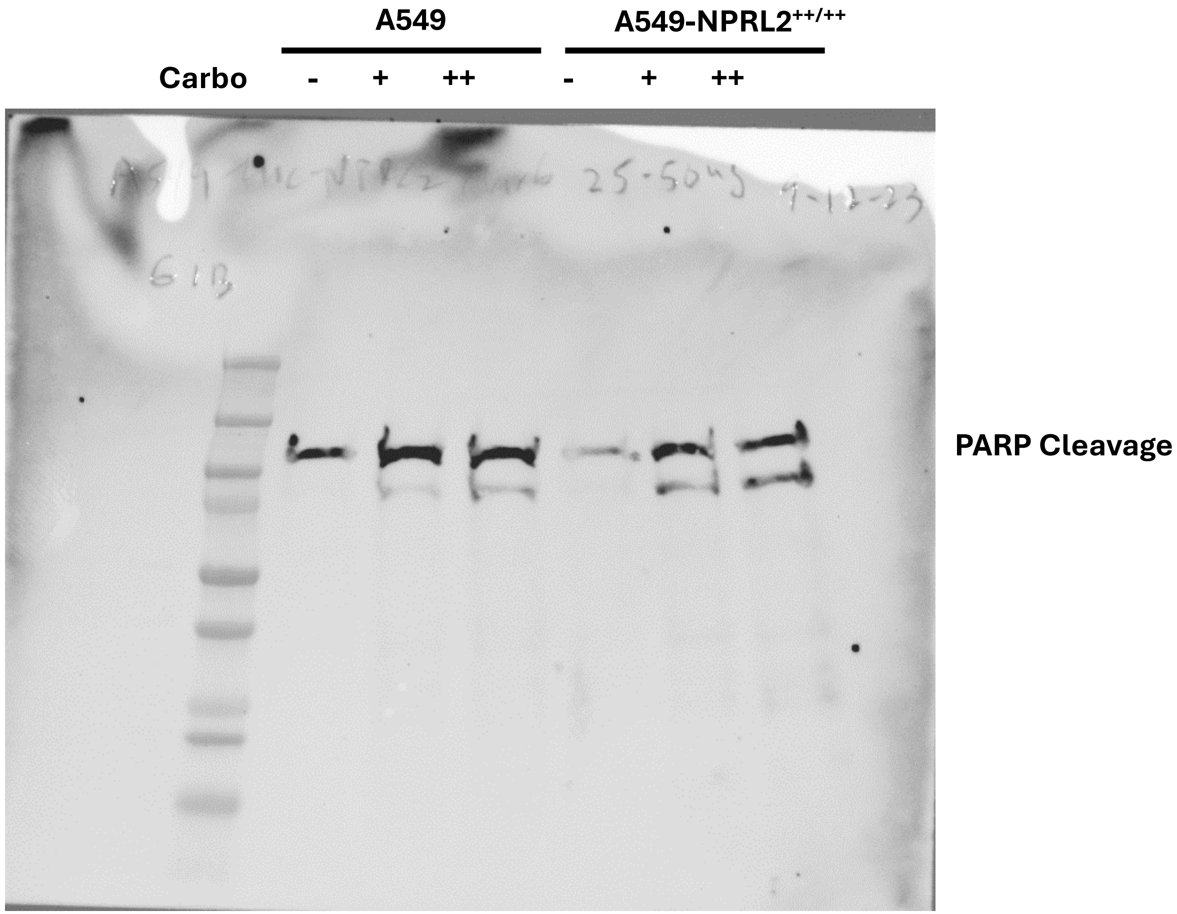

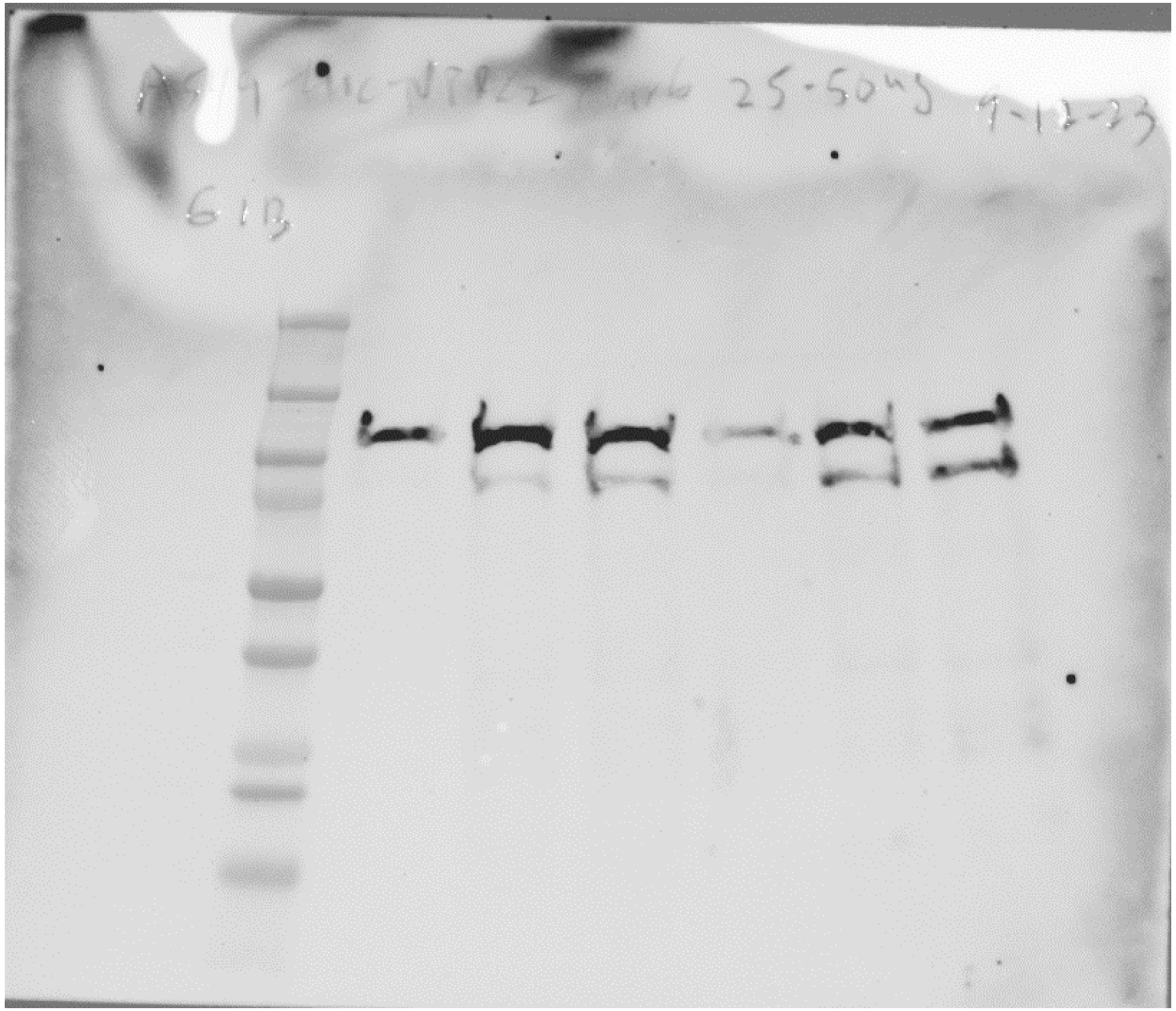

Figure 8-source data 1: PDF file containing original western blots for Figure 8R, indicating the relevant bands and samples

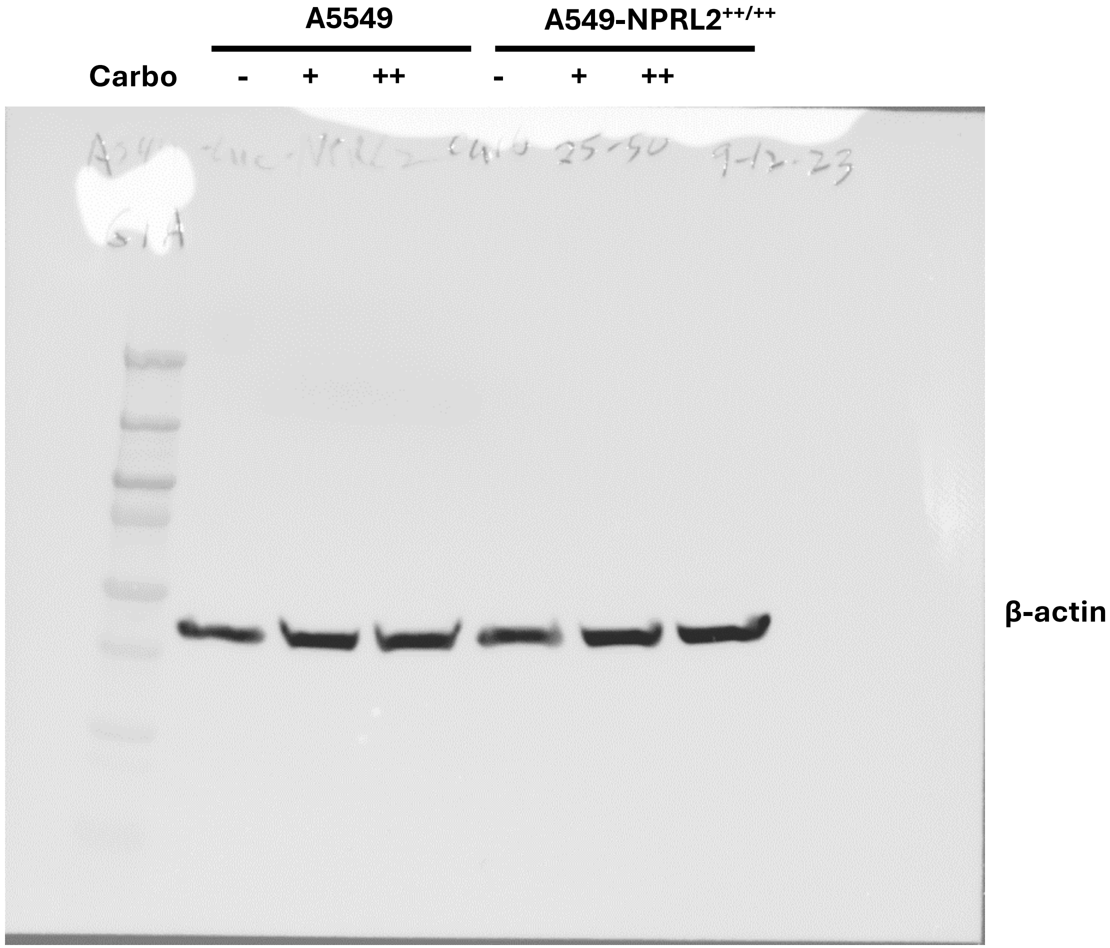

Figure 8-source data 1: PDF file containing original western blots for Figure 8R, indicating the relevant bands and samples

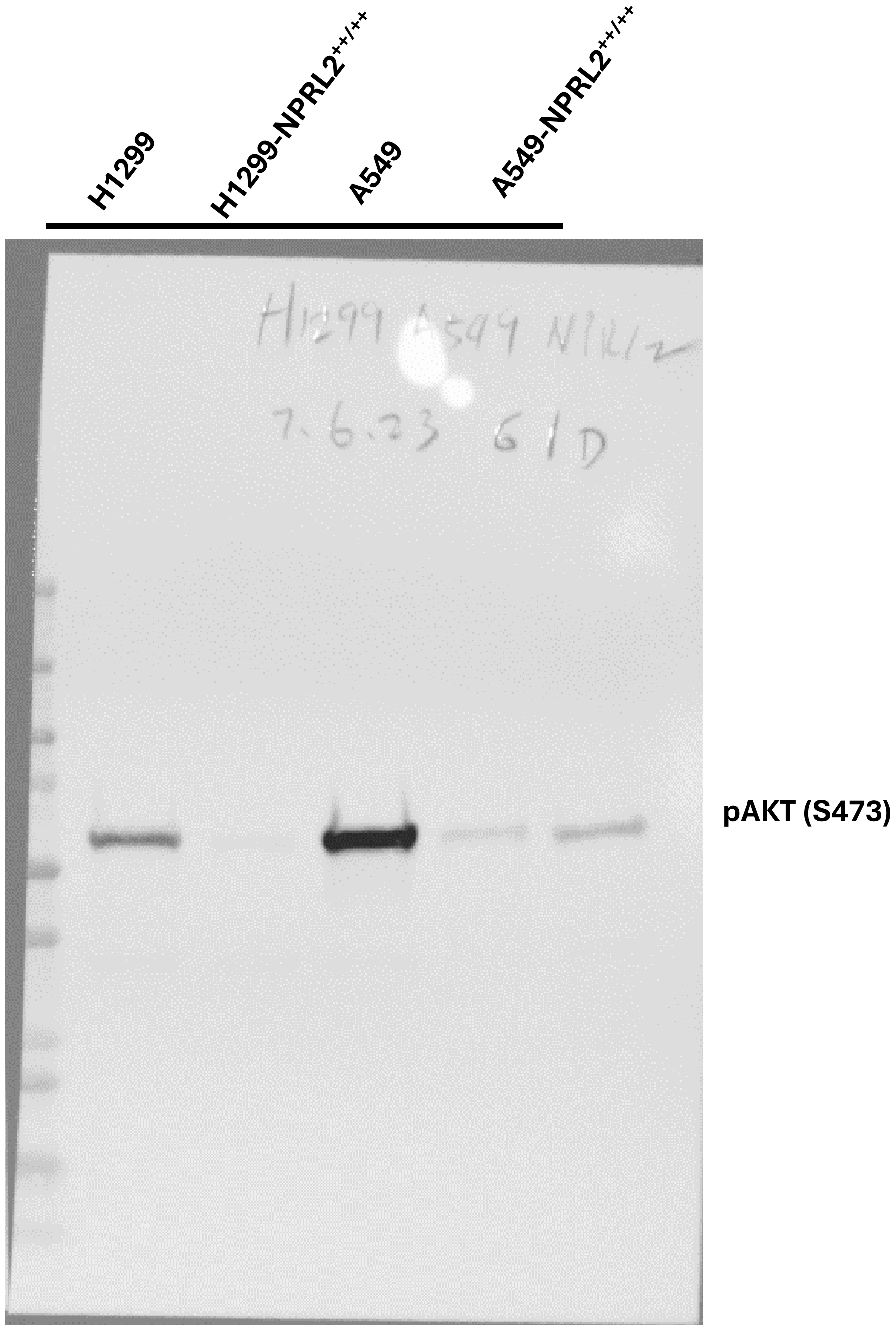

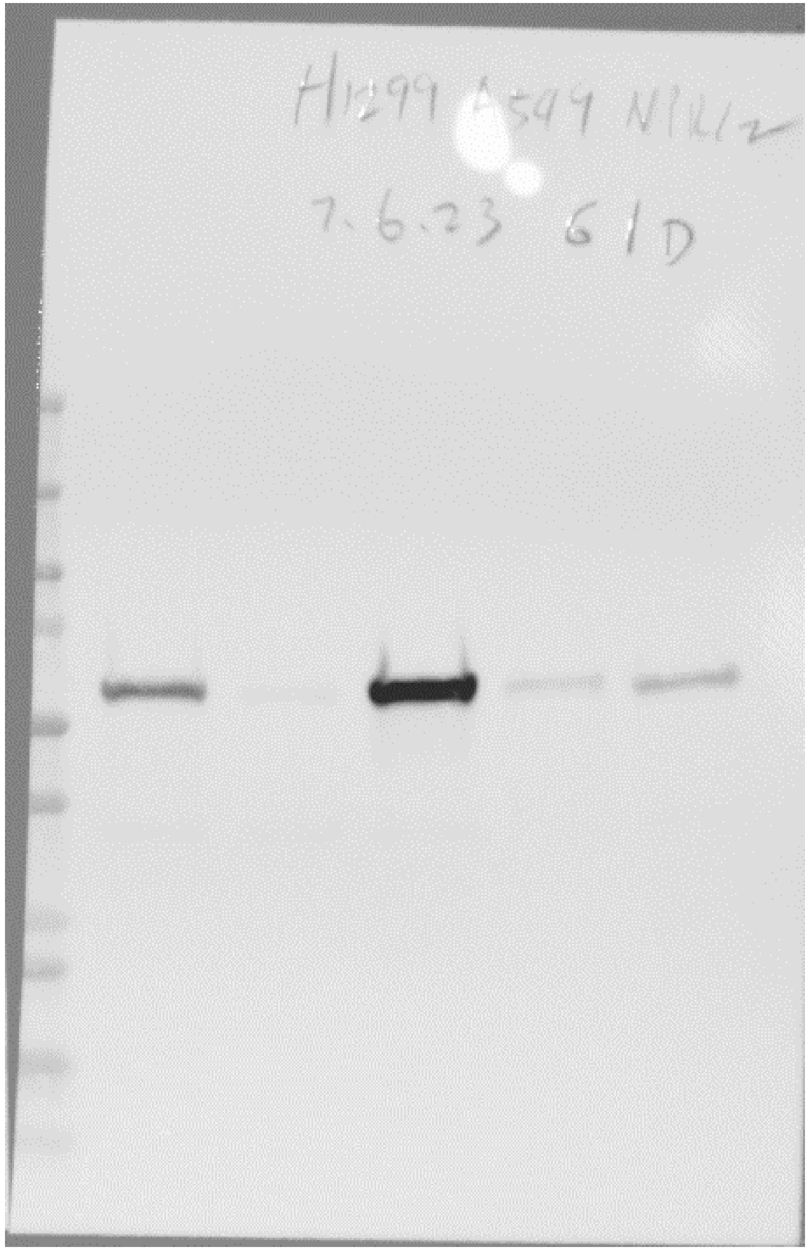

Figure 8-source data 1: PDF file containing original western blots for Figure 8S, indicating the relevant bands and samples

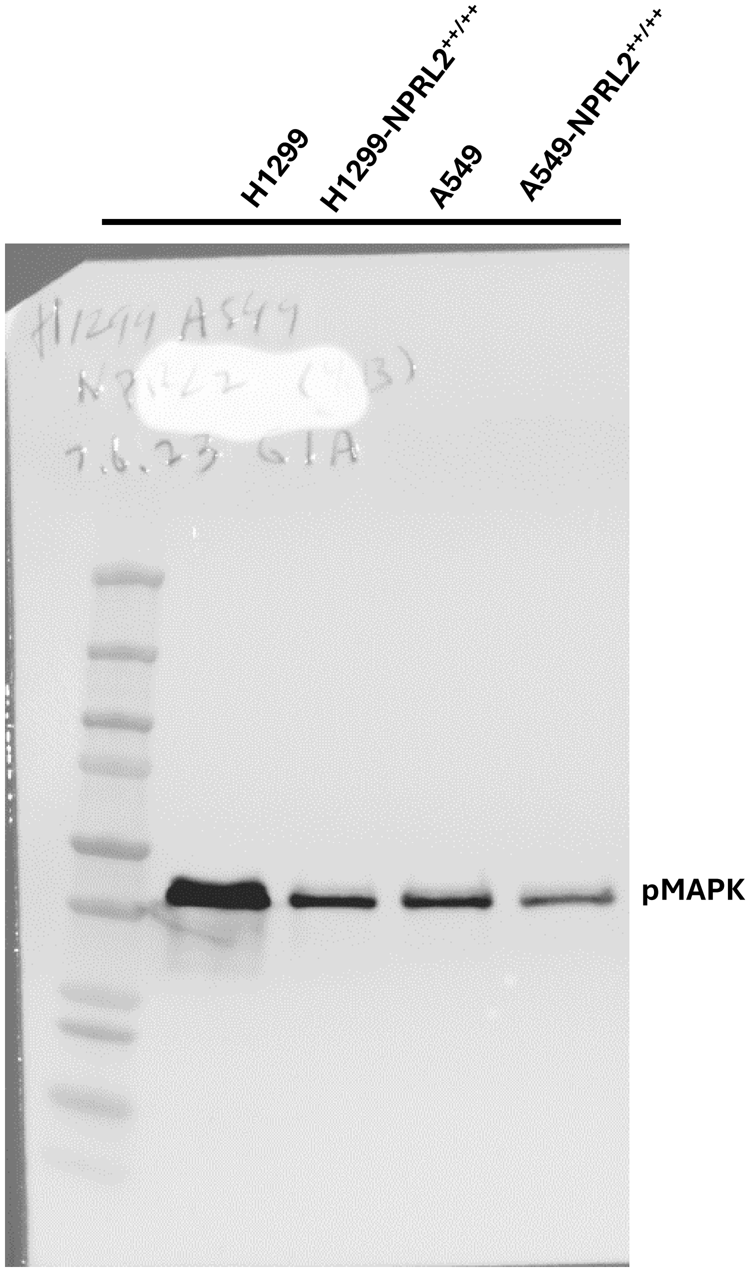

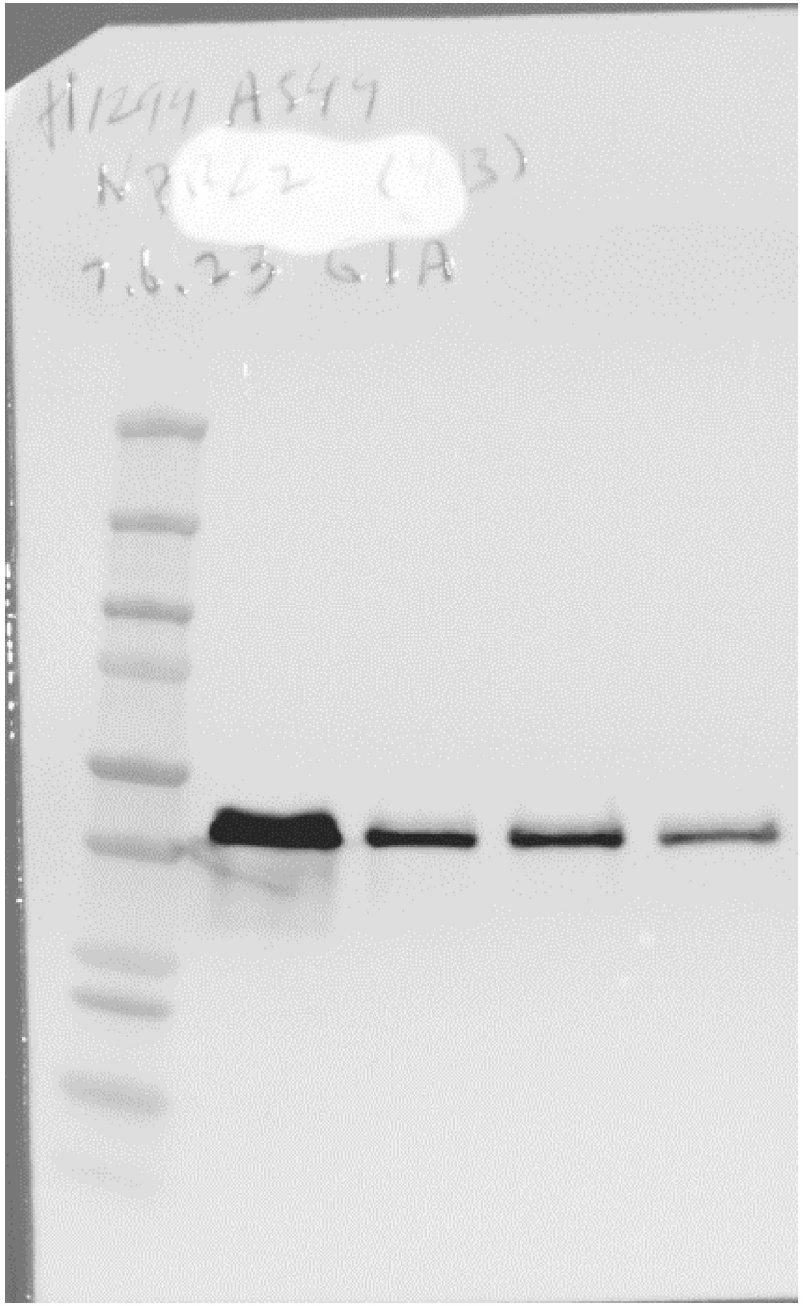

Figure 8-source data 1: PDF file containing original western blots for Figure 8S, indicating the relevant bands and samples

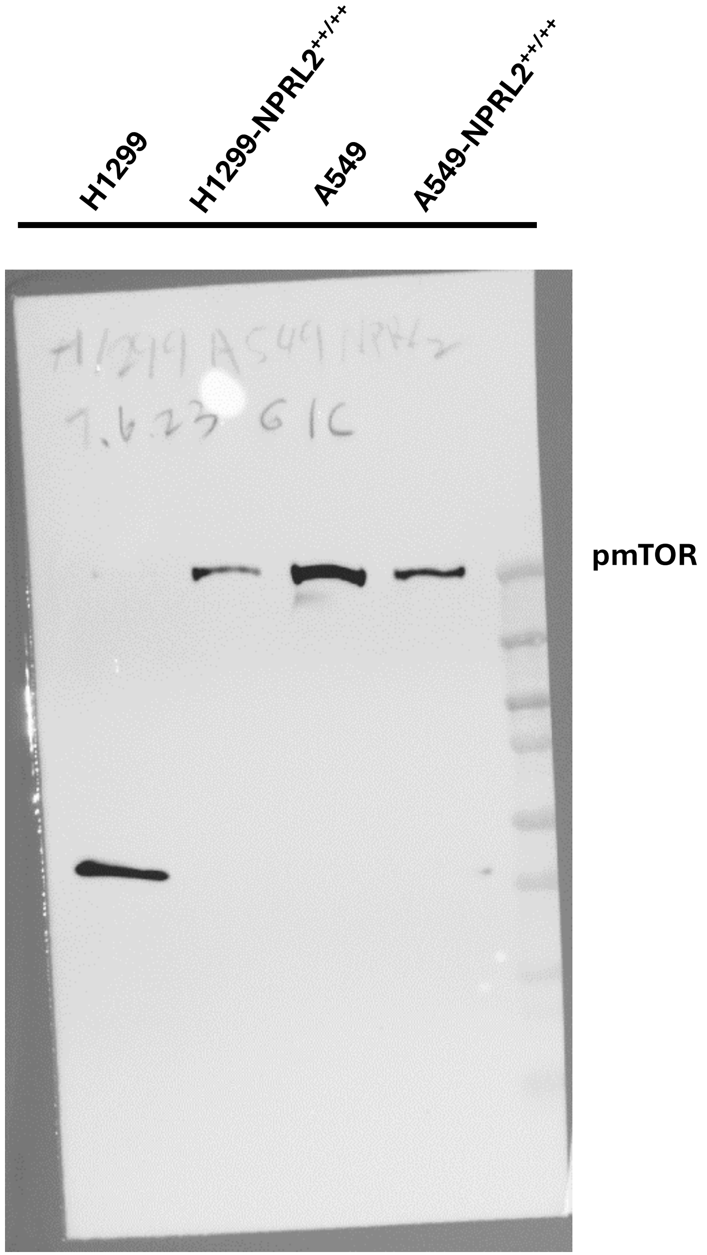

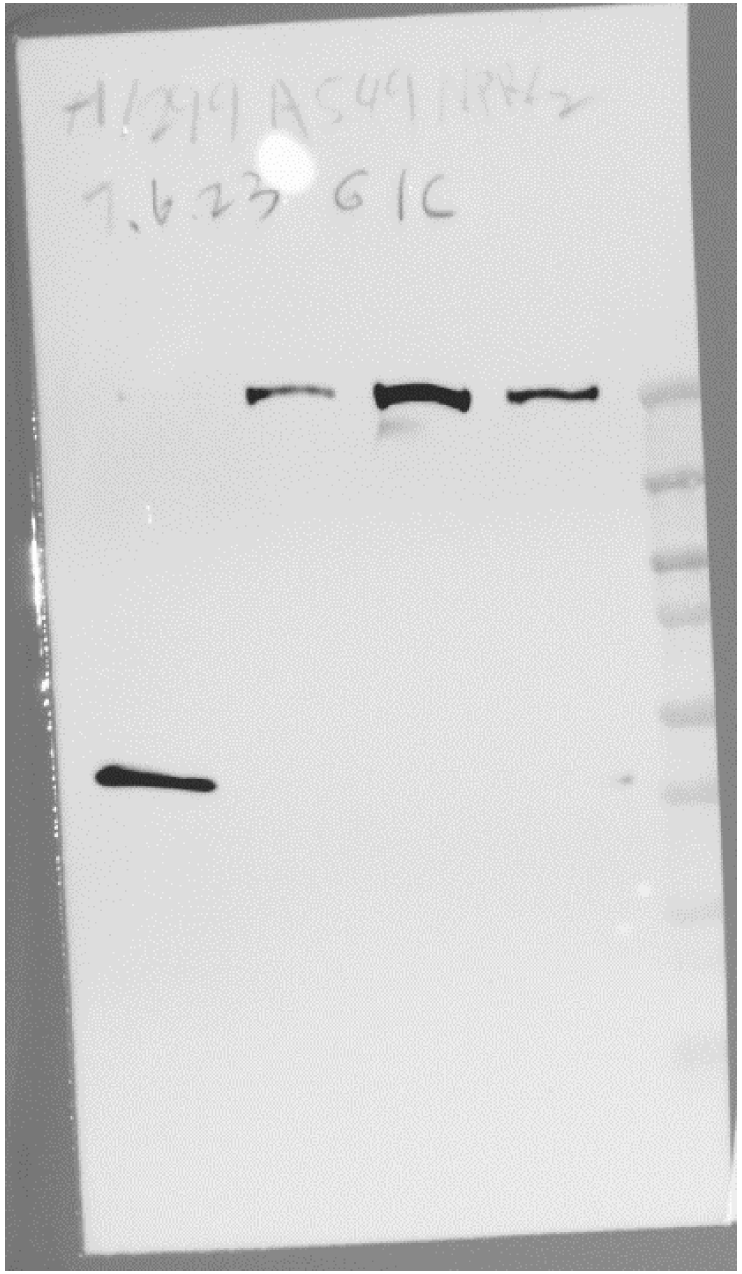

Figure 8-source data 1: PDF file containing original western blots for Figure 8S, indicating the relevant bands and samples

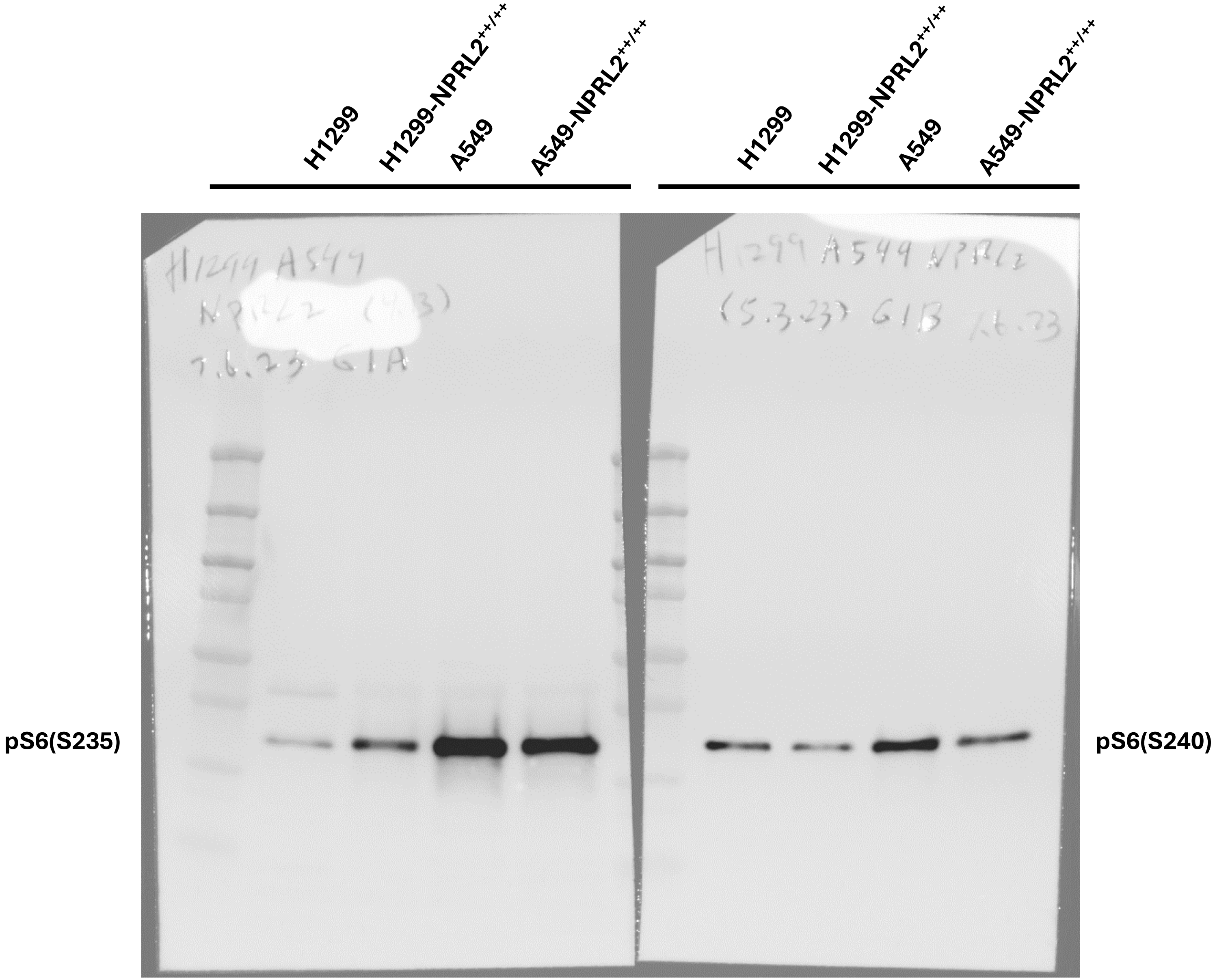

Figure 8-source data 1: PDF file containing original western blots for Figure 8S, indicating the relevant bands and samples

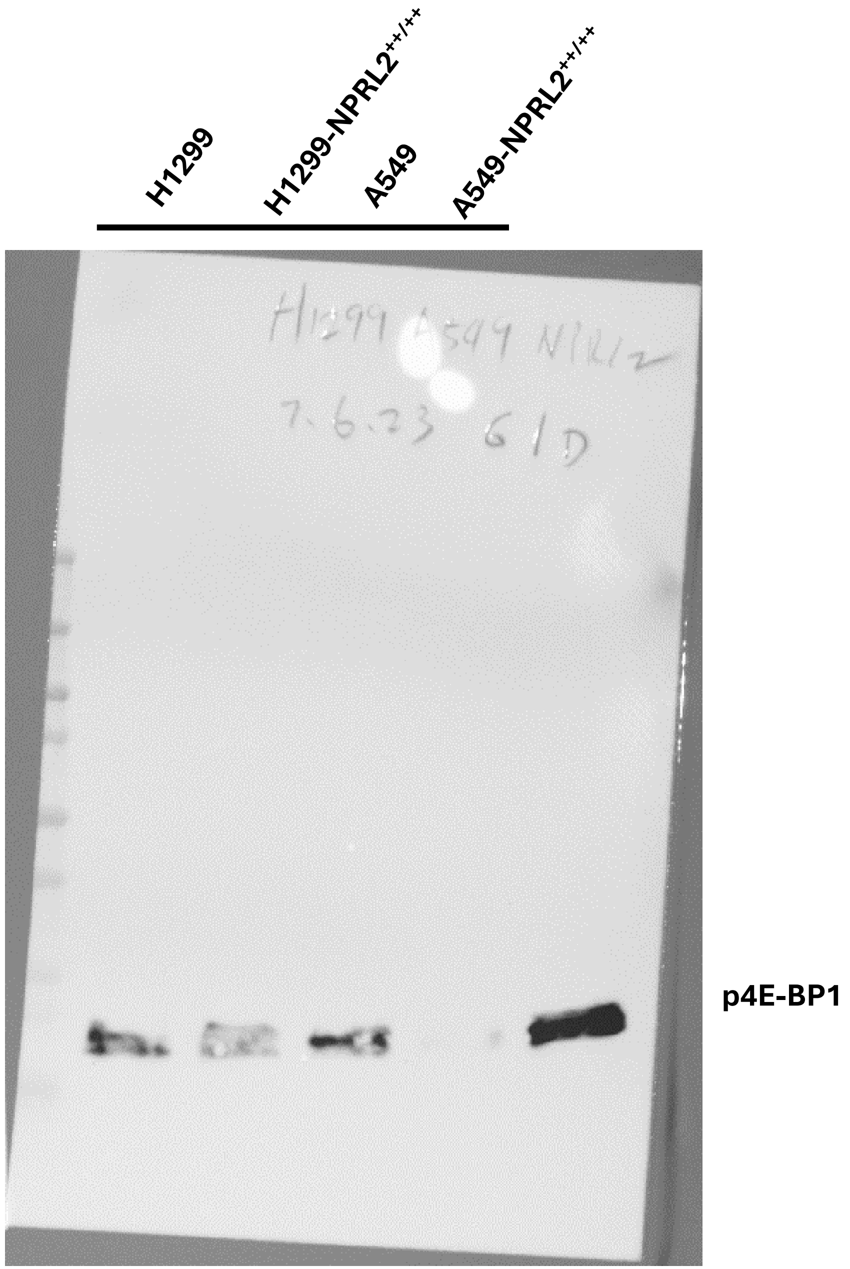

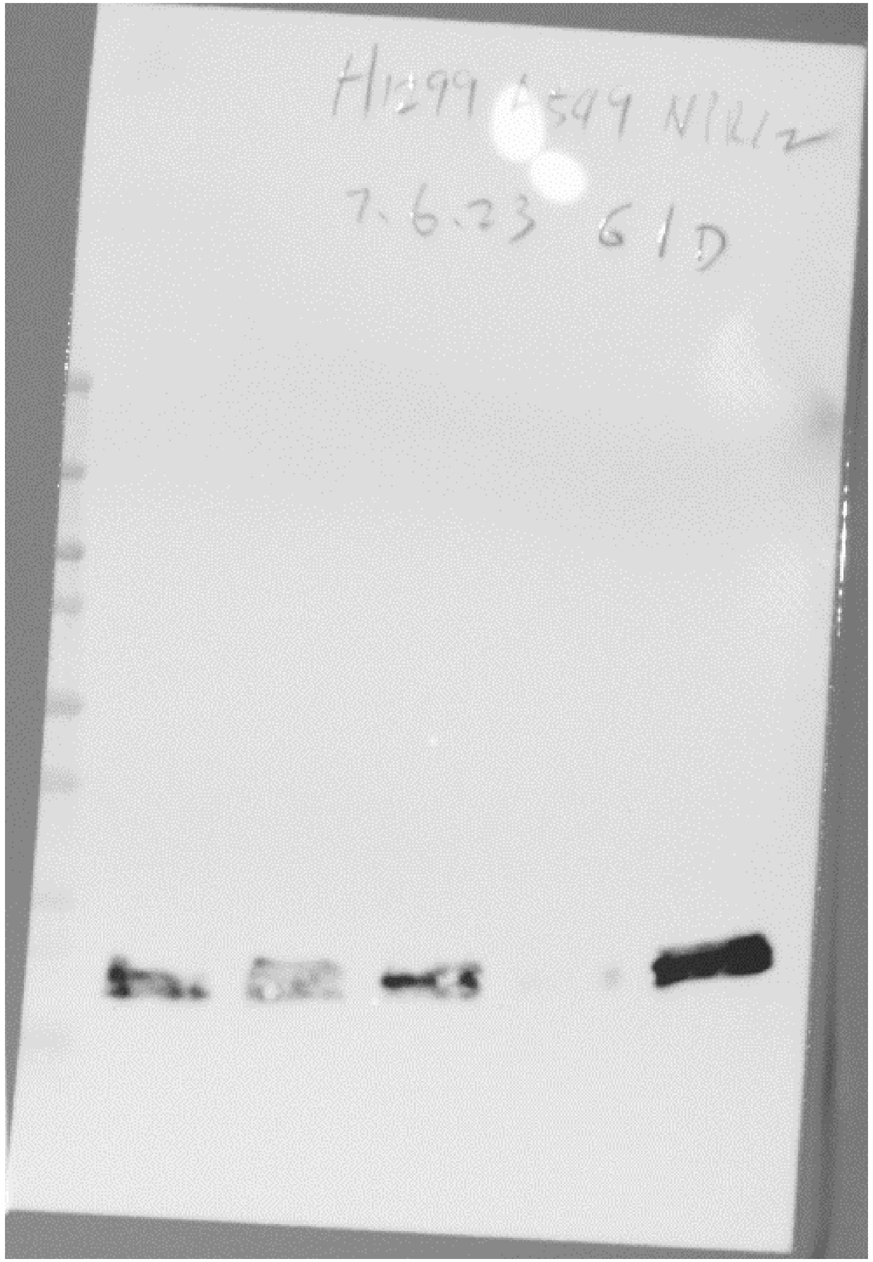

Figure 8-source data 1: PDF file containing original western blots for Figure 8S, indicating the relevant bands and samples

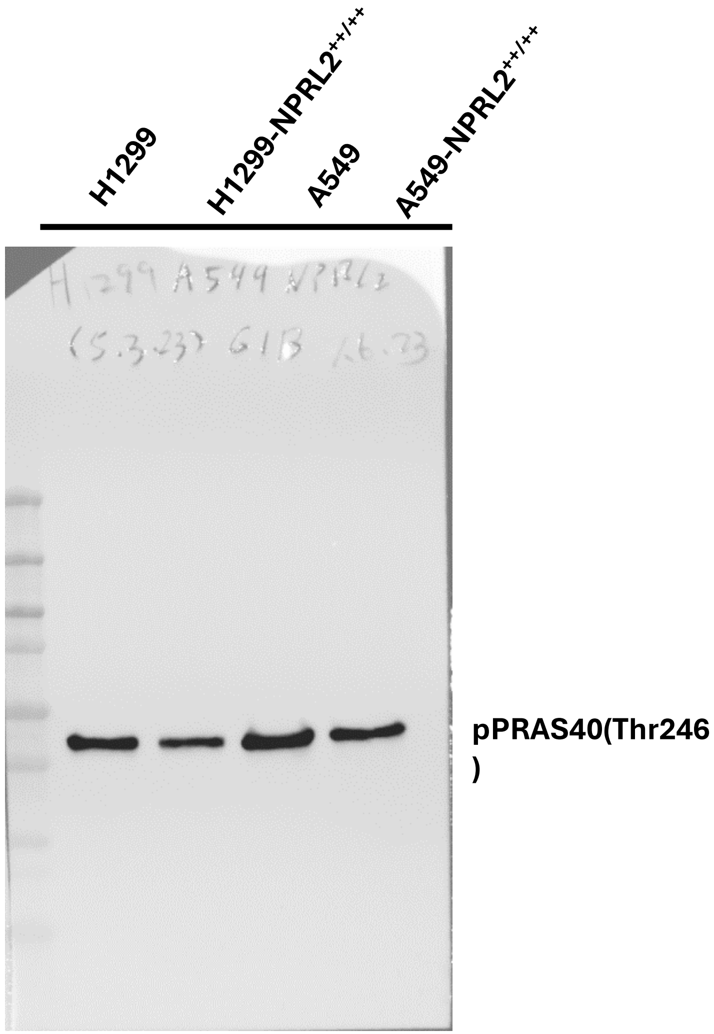

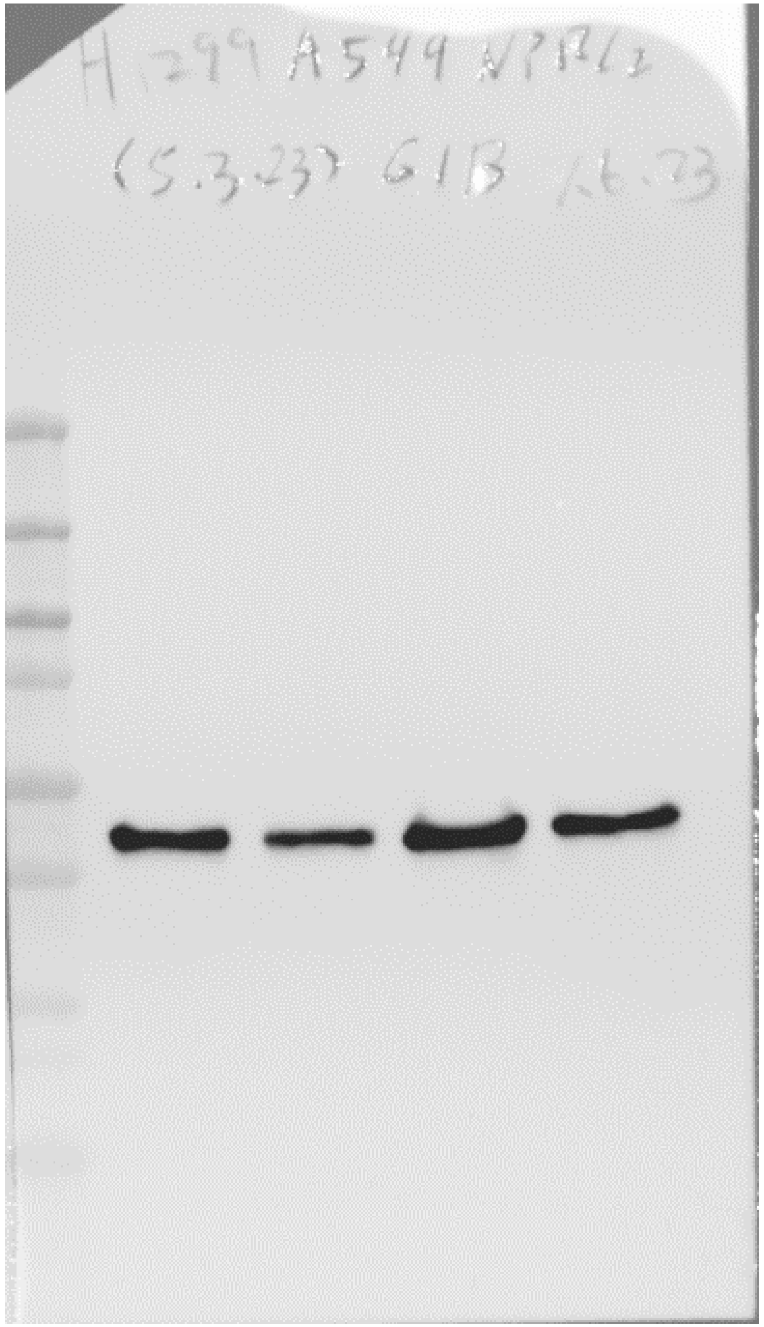

Figure 8-source data 1: PDF file containing original western blots for Figure 8S, indicating the relevant bands and samples

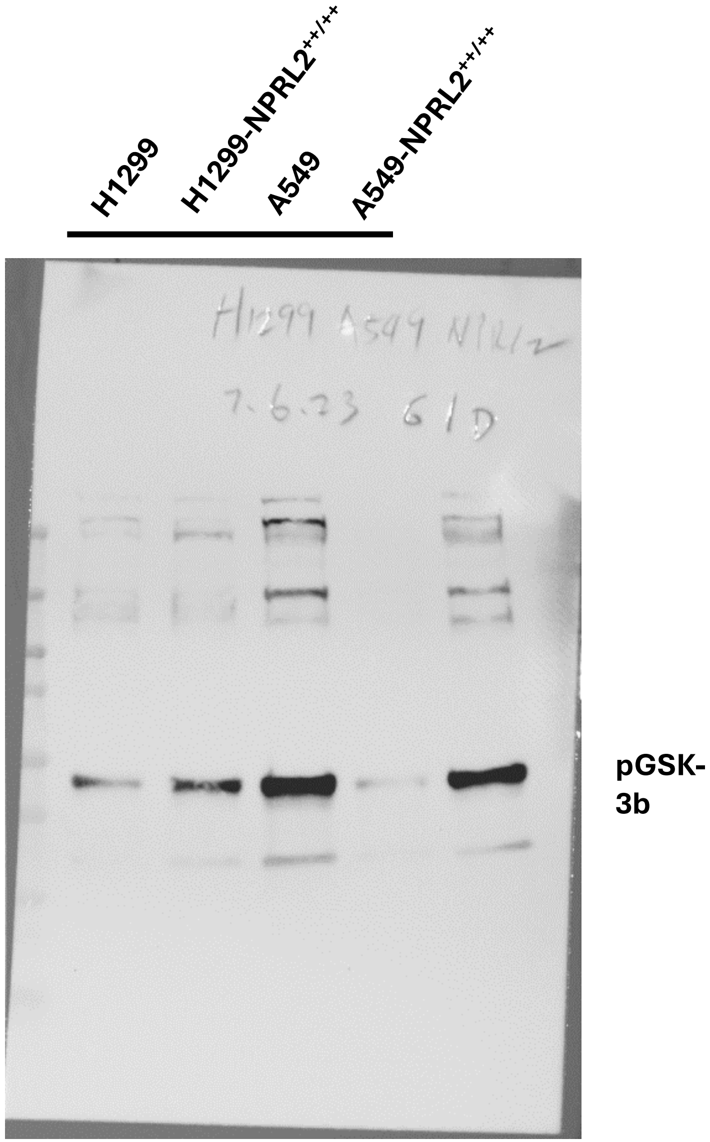

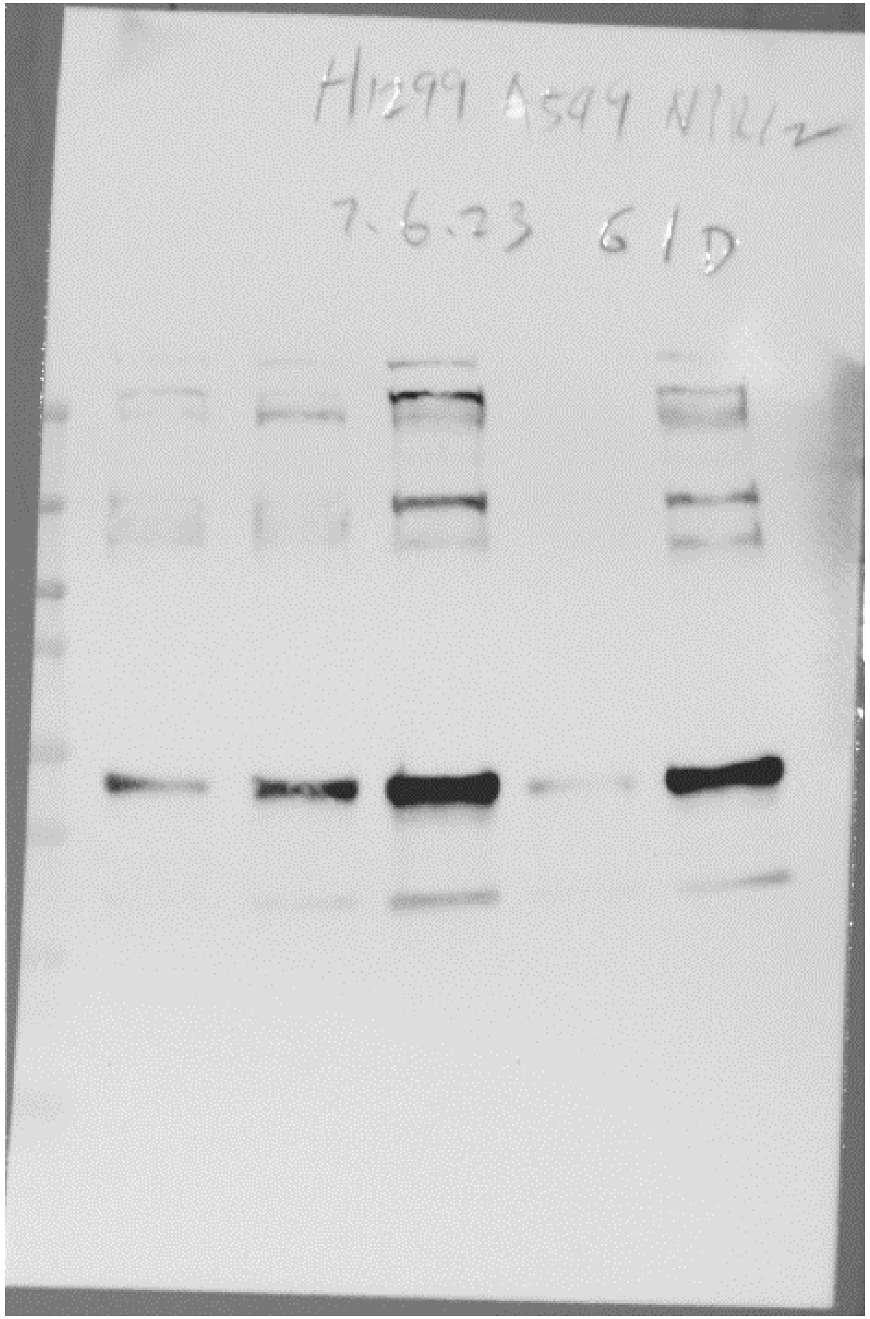

Figure 8-source data 1: PDF file containing original western blots for Figure 8S, indicating the relevant bands and samples

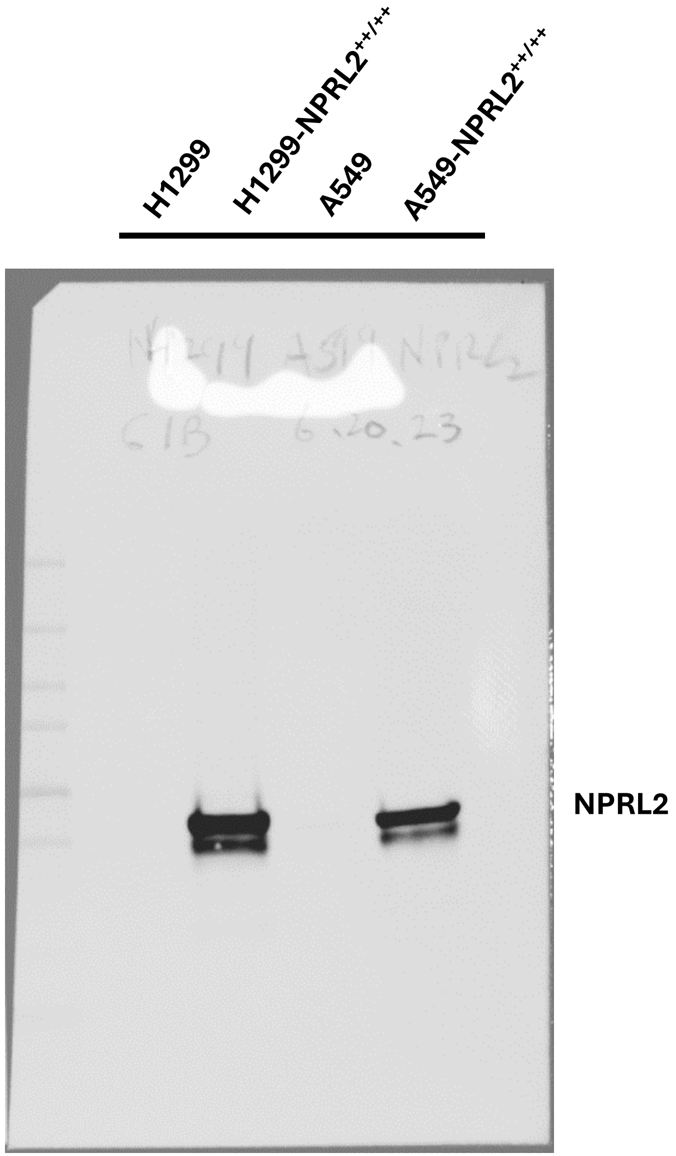

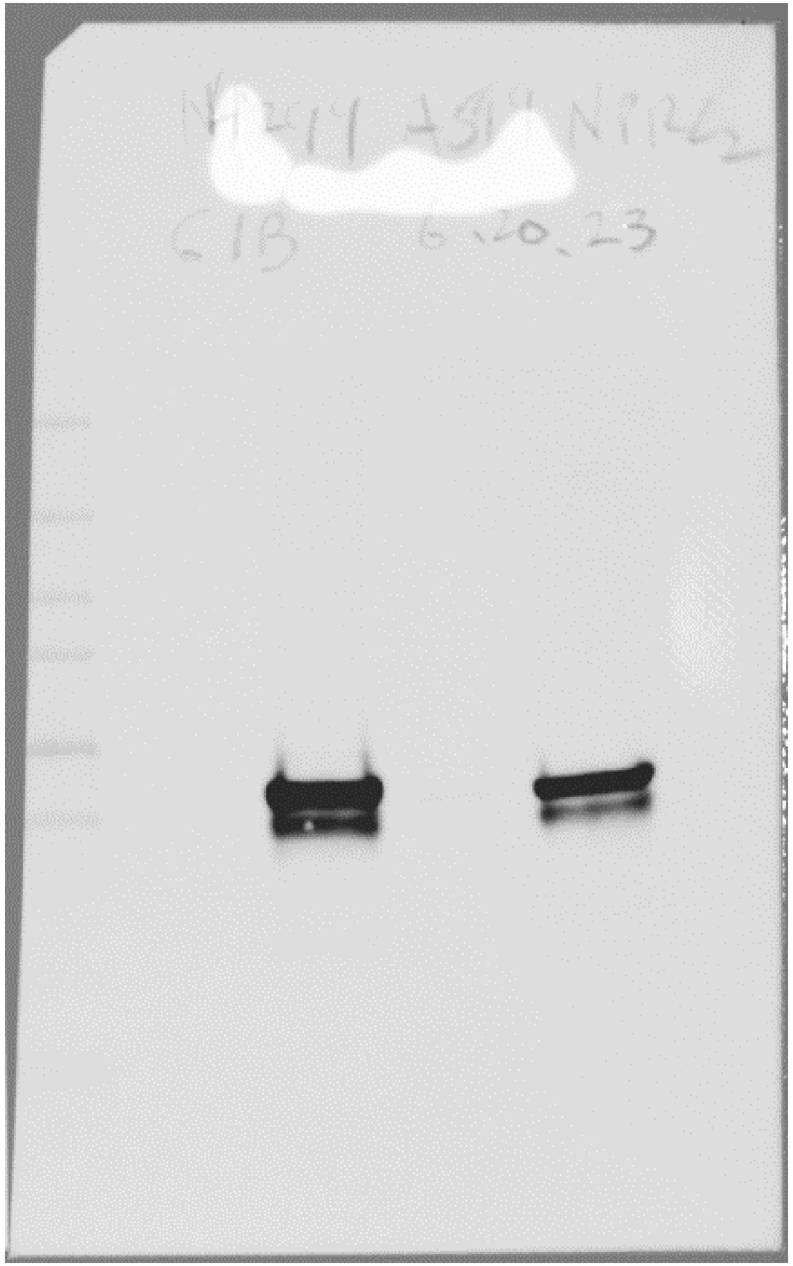

Figure 8-source data 1: PDF file containing original western blots for Figure 8S, indicating the relevant bands and samples

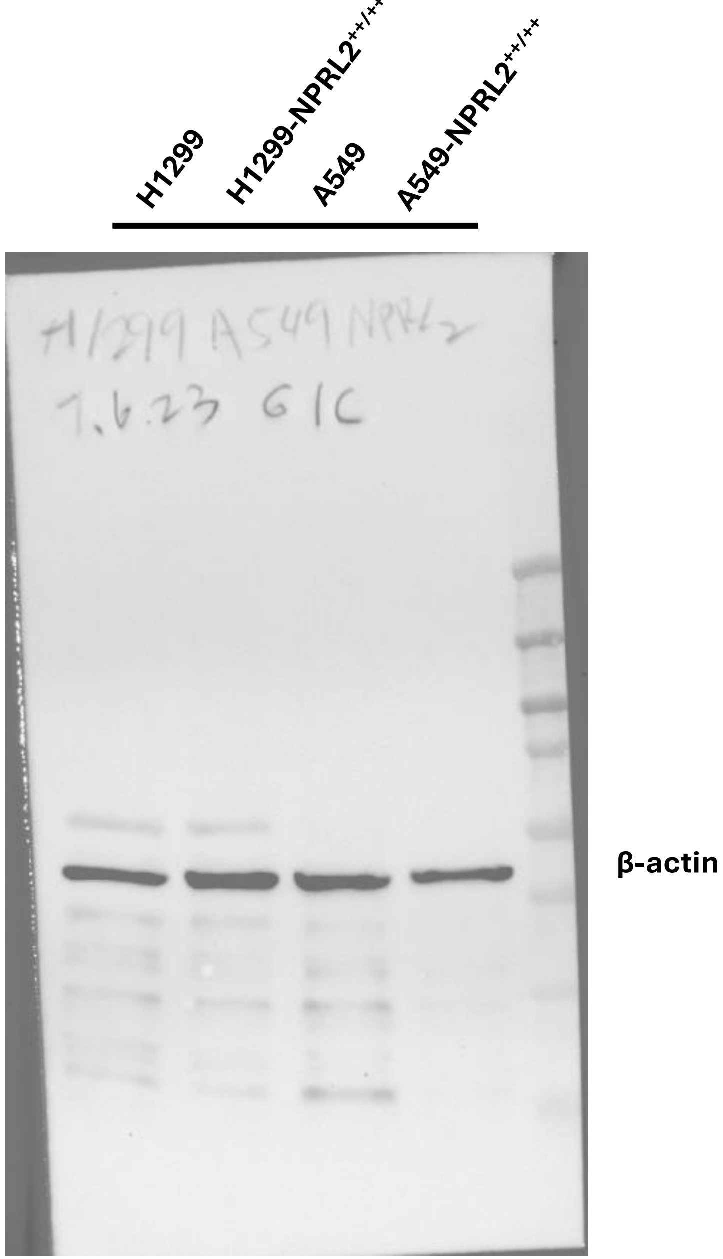

Figure 8-source data 1: PDF file containing original western blots for Figure 8S, indicating the relevant bands and samples
